# Broad range flavonoid profiling by LC/MS of soybean genotypes contrasting for resistance to *Anticarsia gemmatalis *(Lepidoptera: Noctuidae)

**DOI:** 10.1371/journal.pone.0205010

**Published:** 2018-10-03

**Authors:** Jenny D. Gómez, Camilo E. Vital, Maria G. A. Oliveira, Humberto J. O. Ramos

**Affiliations:** 1 Department of Biochemistry and Molecular Biology, UFV, Laboratory of Enzymology, Biochemistry of Proteins and Peptides, BIOAGRO/INCT-IPP, Viçosa-MG, Brazil; 2 Center of Analysis of Biomolecules, NuBioMol, Universidade Federal de Viçosa, Viçosa-MG, Brazil; College of Agricultural Sciences, UNITED STATES

## Abstract

Attack by herbivores is a major biotic stress limiting the soybean crop production. Plant defenses against caterpillars include the production of secondary metabolites such as flavonoids, which constitute a diverse group of plant secondary metabolites. Thus, a more discriminate metabolic profiling between genotypes are important for a more comprehensive and reliable characterization of soybean resistance. Therefore, in this study a non-targeted LC/MS-based for analysis of flavonoid profiles of soybean genotypes contrasting to the resistance to *A*. *gemmatalis* was applied. Clustering analysis revealed profiles highly distinct between the susceptible UFV 105 AP and the resistant IAC 17 genotypes. This comparative approach enables to identify directly from leaf extract some new compounds related to resistance, some of which were present in higher abundance specifically in the IAC 17 genotype: four Quercetin conjugates, Rutin (Quercetin 3-O-Rutinoside), Quercetin-3,7-O- di-glucoside, Quercetin-3-*O*-rhamnosylglycoside-7-*O*-glucoside and Quercetin-3-O-rhamnopyranosyl-glucopyranoside-rhamnopyranoside; two Genistein conjugates, Genistein-7-O-diglucoside-dimalonylated and Genistein-7-O-6-O-malonylglucoside; and one Daidzein conjugate, Daidzein-7-O-Glucoside-malonate. The most abundant flavonoid glycoconjugates in soybean leaves belongs to Quercetin and Kaempferol classes. However, only one from the identified compounds was classified as a Kaempferol. The Kaempferol-3-O-L-rhamnopyranosyl-glucopyranoside showed high abundance in the resistant genotype IAC 17. The metabolic profiles generated by LC/MS allowed the reconstruction of the flavonoid biosynthetic pathways, which revealed a constitutive character for herbivory resistance in the resistant genotype IAC-17 and a metabolic regulation for the rechanneling of Quercetin, Kaempferol and Genistein conjugates in soybean. Highest relative abundances were detected for glyconjugates, such as Rutin, Quercetin 3-*O*-rhamnosylglycoside-7-*O*-glucoside and Quercitin-3-O-rhamnopyranosyl-glucopyranoside-rhamnopyranoside in the leaves of the resistant genotype.

## Introduction

Soybean (*Glycine max* (L.) Merrill) is one of the most important crops in the Brazil, which places the country as the second largest producer in the world. Biotic and abiotic factors can affect the development of this crop, leading to considerable economic losses [1]. *Anticarsia gemmatalis* Hubner 1818 (Noctuidae: Lepidoptera) is one of the major soybean pests in the western hemisphere, including Brazil [2]. This soybean caterpillar is a defoliation pest, consuming leaves during all its larval instars, and consequently can cause complete defoliation of plants [3], diminishing its productivity [2]. Therefore, the development of tolerant genotypes to minimize leaves damage, reducing losses in the productivity is critical. Soybean genotypes with different levels of pest resistance have been developed through conventional genetic breeding [3,4]. Some examples of resistant genotypes developed are IAC 100, IAC 17 and IAC 19 [3]. However, the genetic and molecular mechanisms of resistance have not been evaluated.

Plants respond to herbivory through various morphological, biochemical, and molecular mechanisms to counter or offset the damage effects. The biochemical mechanisms of defense against herbivores are mediated by direct and indirect defenses [5]. Direct acting compounds are either produced constitutively or in response to plant damage, and affect feeding, growth, and survival of herbivores [6,7]. Among these, many phenolic compounds has been characterized as negative agents for herbivores development and survival [5]. In this context, flavonoids play a central role in the plant defense against biotic and abiotic stresses. Flavonoids are divided into various classes; flavonols, flavones and isoflavonoids, which have been investigated as feeding deterrents against insect pests, including *A*. *gemmatalis*, *Spodoptera frugiperda* and *Piezodorus guildinii* [3,7–11].

Qualitative and quantitative profiling of flavonoids in plant extracts is a complex task because these comprise a large group of structurally diverse analyses, and formed a variety of core compounds (aglycones), that mostly occur in plants in the form of glycoconjugates (the -OH groups of flavonoid aglycones are substituted with various saccharides). Consequently, numerous flavonoid aglycones are glycosylated at multiple sites with a variety of saccharides, thus producing several thousands of compounds chemically distinguishable [12]. Thus, flavonoids profile analyses by HPLC or LC/MS-based traditional methods could not explore the full potential complexity of the biosynthetic pathways responding to stress [13].

Although characterized as mediators of the direct defense, plant metabolic and signaling pathways of flavonoids have been under characterization [10,12,14–16]. However, some studies have showed that the overexpression of transcription factors designed as PFG1–3 (*Production of Flavonol Glycosides*) and MYB75 controlling flavonoid production in *Arabidopsis* plants conferred resistance to *Pieris brassicae* caterpillars [10,15]. Thus, studies on these secondary metabolites could lead to the identification of new signaling molecules involved in plant resistance against herbivores and other stresses. Eventually, genes and enzymes involved in the biosynthesis of these metabolites could be identified. Therefore, in this study a non-targeted method LC/MS-based [13] was applied to perform flavonoid profile analysis in two soybean genotypes contrasting for herbivory resistance in response to *A*. *gemmatalis*. This broad range approach enabled to access more complex profile directly from soybean leaf extract and efficiently identifying some new resistance related compounds. The metabolic profiles enable the reconstruction of the flavonoids biosynthetic pathways, revealing their abundance differences between genotypes contrasting for herbivory resistance. This could reflect the differences in the genetic background and metabolic regulations.

## Materials and methods

### Plant growth and soybean genotypes

Soybean plants were cultivated in greenhouse and maintained isolated in cages and without addition of agrochemicals during assays. The cultivar UFV TN 105 AP designated as “105 AP” is a variety of early cycle that was developed by the Soybean Breeding Program of the Biotechnology Institute of the Universidade Federal de Viçosa (BIOAGRO/UFV). In this genotype, three isoforms coding for LOX1, LOX2 e LOX3 lipoxygenases were eliminated [12, 17]. The cultivar IAC 17 is a variety of early cycle [18–20], of genealogy D 72-9601-1 x ‘IAC 8’ and considered a resistant genotype to herbivory against *A*. *gemmatalis*, evaluated for caterpillars fed soybean leaves at greenhouse conditions [3,21,22]. The resistances were evaluated by the Kaplan-Meier method [23]. Seeds of each variety were selected and submitted to pre-germination until reach 0.5–1 cm radicle size. Germinated seeds were carefully transplanted to vessels containing 2.0 kg of a mixture of soil, sand and dung (3:1:1). Soybean plants were irrigated daily, and kept for 30 days at greenhouse conditions of 25 ± 5° C and 70 ± 10% of relative humidity.

### Insects

A laboratory colony of *A*. *gemmatalis* was started with eggs obtained from the National Research Center of Soybean (CNP–Soja, EMBRAPA, Londrina, PR, Brazil). The insects were reared on artificial diet and maintained under controlled conditions of 25 ± 5°C, 70 ± 10% RH and 14:10 (L:D) photoperiod. *Anticarsia gemmatalis* adults were kept in cages (50 x 50 cm) with paper sheets for oviposition, and fed with nutrient solution with honey (10.5 g), beer (350 mL), sucrose (60 g), ascorbic acid (1.05 g), nipagin (1.05 g), and water (1050 ml), embedded in cotton placed at the bottom of the cages in a Petri dish. *Anticarsia gemmatalis* egg masses were collected every three days and first instar larvae transferred to plants. After eclosion of the larvae, the first instar caterpillars were transferred to the respective soybean genotype used in the assays.

### Evaluation of *A*. *gemmatalis* survival

Ten soybean plants from each 105 AP and IAC 17 genotypes at V4 or V5 developmental stage were infested with 400 first instar *A*. *gemmatalis* caterpillars (20 caterpillars per plant). Plants were kept in the laboratory at 25 ± 5°C and 60 ± 10% RH, protected by insulated and sanitized containers to avoid having other factors that could affect the larvae welfare. Larvae survival feeding each plant genotype was monitored daily during 15 days and estimated by the Kaplan-Meier method [23]. Survival curves were compared by the Log-Rank test [23].

### Caterpillar infestation assays and leaves extract preparation

Experiments were carried out using 40 plants, grouped in four blocks, from each soybean genotypes 105 AP and IAC 17 at V4 or V5 developmental stage. Two leaves per plant were isolated with plastic cages and were infested with 20 first instar *A*. *gemmatalis* caterpillars. A plant group from each genotype were kept as control and did not receive the caterpillars. Plants from each block and treatment were combined to compose pools, which were used as four biological replicate. After 48 h, leaves were collected from control (not infested) and infested plants and stored in freezer at -80°C.

Leaves extract was prepared by grounding 100 mg of leaves, from pools containing 10 plants, in liquid nitrogen and for each sample 200 ul of extractive solution (75% methanol/ 0,1% formic acid) were added [24–25]. After that, samples were subjected to ultrasound treatment for 30 min, followed of centrifugation at 14.000*g*, for 30 min, at 4°C. The supernatant was collected, and the procedure repeated twice. The methanolic plant extracts were lyophilized, ressuspended in water and stored freezer at -80°C until biochemical analyzes were performed.

### Strategy for flavonoids profiling by LC/MS

Aliquots of 300 uL of the extracts were placed in vials and 5 uL injected into the LC/MS system in the NuBioMol (Center for Biomolecules Analysis-UFV, Brazil). For all LC/MS analysis were used an Ultra High Performance Liquid Chromatography (UHPLC; model 1200 Infinity) with a chromatography column (Agilent Eclipse Plus, RRHD, 1.8 um, 2.1x50 mm) and a flow of 0.3 mL/min, coupled online to a mass spectrometer QQQ (triple quadrupole; Agilent6430). Chromatographic separation was carried out on a column Zorbax Eclipse Plus C18 (1.8 μm, 2.1 x 50mm) (Agilent) and a guard column Zorbax SB-C18, 1.8 μm (Agilent). The mobile phase consisted of buffers (A) water acetic acid 0,02% and (B) acetonitrile acetic acid 0,02% (LC/MS grade from Sigma-Aldrich). A gradient of %B de: 5%/0 min; 60%/11 min; 95%/13 min; 95%/17min; 5%/19 min; 5%/20 min was applied. The solvent flow rate was 0.3ml/min in a column, at 30°C. The mass spectrometer operated by positive mode according to the method for flavonoids detection. Analyses were carried out for four pools as biological replicates, containing 10 plants each.

**Fig 1:**
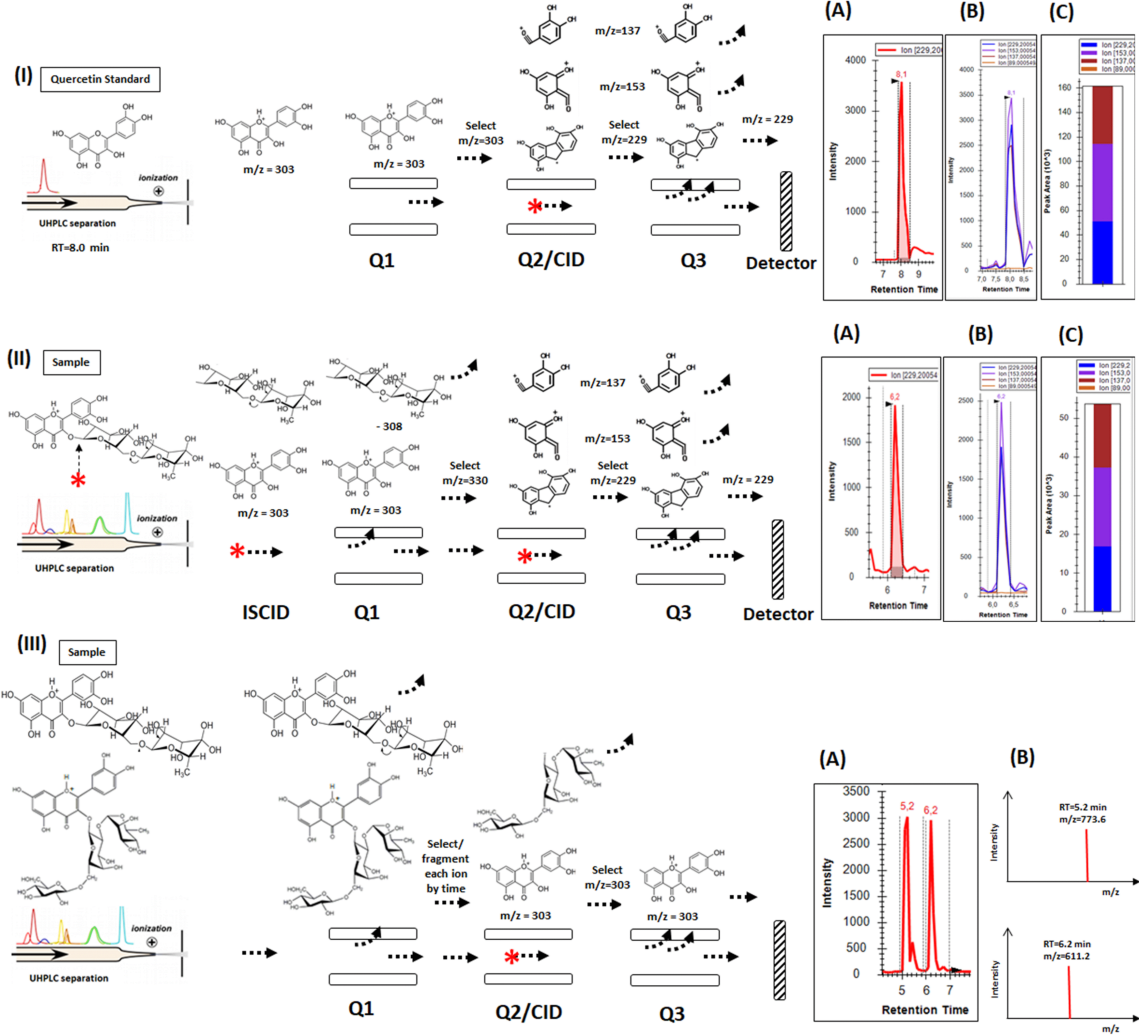
Schematic illustrations of the mass spectrometry approaches used for flavonoid profiling from soybean leaf. In **(I)** each standard aglycone flavonoid is injected in LC QqQ mass spectrometer, a *product scan* method is applied for determination of relative intensities of the four discriminated fragments, which will be used to generate the RFI% specific for each class in (**C**). The area from each MRM transition is processed using Skyline package in **(A)**, **(B)** and **(C)**. In **(II)** a non-target method is applied to the samples to enable the detection of all flavonoids compound containing each aglycone class. A in source activation ISCID is used for fragment the glycoconjugate and to release the aglycone core, which is selected in first quadrupole (**Q1),** fragmented at the second (Q2/CID) and the product ions are monitored be four MRM transition for the ions that discriminate for each class determinate at the method describe in **(I)**. In the example of the figure the compound ionizing by ESI release a specific mass of m/z = 303, which RFI% generated (**A, B** and **C**) share high similarity with the standard Quercetin (**IC**). Thus, this compound eluted at RT = 6.2 min is characterized as a Quercetin glycoconjugate. In **(III)** a *precursor ion scan* method is applied to verify in the samples all compound having a chemical group with m/z value of 303 Da and generating the four fragments specific for Quercetin aglycone. The XIC generated in (**A** and **B**) are verified for the precursor m/z values in (**C**). Research in the mass spectrum database and literature of the mass of 611.2 Da detected at the RT = 6.2 is indicate of the presence of the Quercetin glycoconjugate Rutin in the samples.

Flavonoid profiles were generated using three consecutive strategies, as illustrated in the Fig 1. First, 21 target phenolic compounds were analyzed (S1 Table). Crystalline reference substances of flavonols (Kaempferol, Quercetin, Myricetin, Catechin, Epicatechin, Rutin and Morin), flavones (Luteolin, Apigenin, Oerientin, Isoorientin, Vitexin, Isovitexin), flavanones (Naringin, Hesperetin, Naringenin and Hesperidin), chalcones (Chalcone and Phloretin) and isoflavones (Daidzein, and Genistein) were obtained from Sigma-Aldrich (St. Luis, MO, USA). A standard curve of each compound, in a concentration range from 1 to 20,000 ng/mL, was used to convert the area values from XICs (*extracted ion chromatogram*) in ng/g of fresh leaf tissue. In this method, the retention times and the MRM (*multiple reaction monitoring*) transitions, generated for each standard, were tabulated to format *transition list* table and was used as input in the Skyline package, enabling quantitative analyses of the specific compounds identified in soybean leaves.

Second strategy involved the use a non-target method in source fragmentation ISCID for the analysis of flavonoid classes [13] with some modifications. The standards were grouped into classes based in the aglycone core. Insource fragmentation was applied for each compound and a *product ion scan* method was used to experimental determination of the fragment ions produced and to select which showed distinguible relative intensity between the classes. Fragments intensities were computed in terms of percentages, as illustrated in the Fig 1 IA, to generate relative proportions (FRI%, *fragment relative intensity*). These were used as signature to identify the presence of compounds for each class. Followed, an insource/MRM method was generated using the representative m/z of each aglycone class and four MRM transition to enable scanning along all the LC/MS run (Fig 1 II). All flavonoids containing different glycoconjugates were linked to each class of the aglycone core. The LC/MS raw data generated were analyzed by Skyline package using as input a *transition list* without retention times (S3 Table), which turns possible the software to compute all the chromatogram pics generated for every MRM transition along of the entire run. The most abundant XIC (*extracted ion chromatogram*) was selected for quantitative profile analysis from four biological replicates.

Finally, as a thirty method, the m/z values were determinate. The ions that generated the XICs were selected in the insource/MRM method. A LC/MS method was created by *precursor ion scan* for the most intensity fragment ion from each flavonoid class (S2 Table). The mass spectra generated were analyzed using the Mass Hunter package (Agilent) and the XICs (Fig 1 IIIA) observed were verified manually for the m/z values of the precursor ion (S1 Fig and Fig 1 IIIB) for those same RT were observed anteriorly for the aglycones core at the pseudo MS3/MRM method. Additionally, the retention times generated by pseudoMS3/MRM and *precursor ion scan* methods were compared to verify the corresponding aglycone and glucoconjugated flavonoid. Each m/z value, for the deprotonate precursor ion observed, were searched against the *Mass Bank* (http://www.massbank.jp), using the *Quick Source* module with exact *mass tolerance* of 0.3 Da. A search was also realized using the flavonoid class name and the nominal mass to identify compounds described in the literature. Additionally, each hit was verified for the flavonoid class, for aglycone mass and for the presence in the MS/MS spectrum of the same fragments used for the generation of the FRI%.

### Metabolic pathways and statistical analysis of flavonoid profiles

The abundance of the each characterized flavonoid from each genotype and treatment was combined and analyzed in the MetaboAnalyst platform (www.metaboanalyst.ca). Quality filters based on the standard deviation method were used to automatic remove low-quality data, then the intensity values were normalized by sum. Data was analyzed using the Partial Least Squares Discriminant Analysis (PLS-DA) to generate 2D Score Plots displaying genotypes and treatments effects on data grouping. A cluster analysis by HeatMap method was also performed to each group flavonoid in accordance to their relative abundances in genotypes and treatments, using the following parameters: Distance Measure: Euclidean; Clustering Algorithm: Ward; Data Source: Normalized Data; Standardization: Autoscale features; T-test/ANOVA; Options: “Show only group averages” and View Mode “Overview”. For quantitative analysis of characterized compounds, the “One-way Analysis of Variance (ANOVA)” was used with the following parameters: Adjusted p-value (FDR) cutoff: 0.05; Post-hoc analysis: Fisher’s LSD.

Finally, the identified compounds were organized in accordance to the metabolic pathways for flavonoid biosynthesis using as reference the *Glycine max* maps from the KEGG (Kyoto Encyclopedia of Genes and Genomes) repository.

### Statistical analysis

The quantitative analysis of the LC/MS data were performed for four replicates. Analysis of variance (ANOVA, p < 0.05) were used to evaluate the effects of the plant genotypes and the presence or absence of the caterpillars. PLSA-DA and cluster analysis were performed using default parameters of the *MetaboAnalyst* plataform. Statistical analysis of the caterpillar survival were evaluated as describe by [23].

## Results

### *Anticarsia gemmatalis* survival on soybean genotypes

Caterpillar survival analysis using the Kaplan-Meier method showed that the final survival percentage was 73% for caterpillars fed with leaves from 105 AP genotype, between 7 and 10 days.

**Fig 2:**
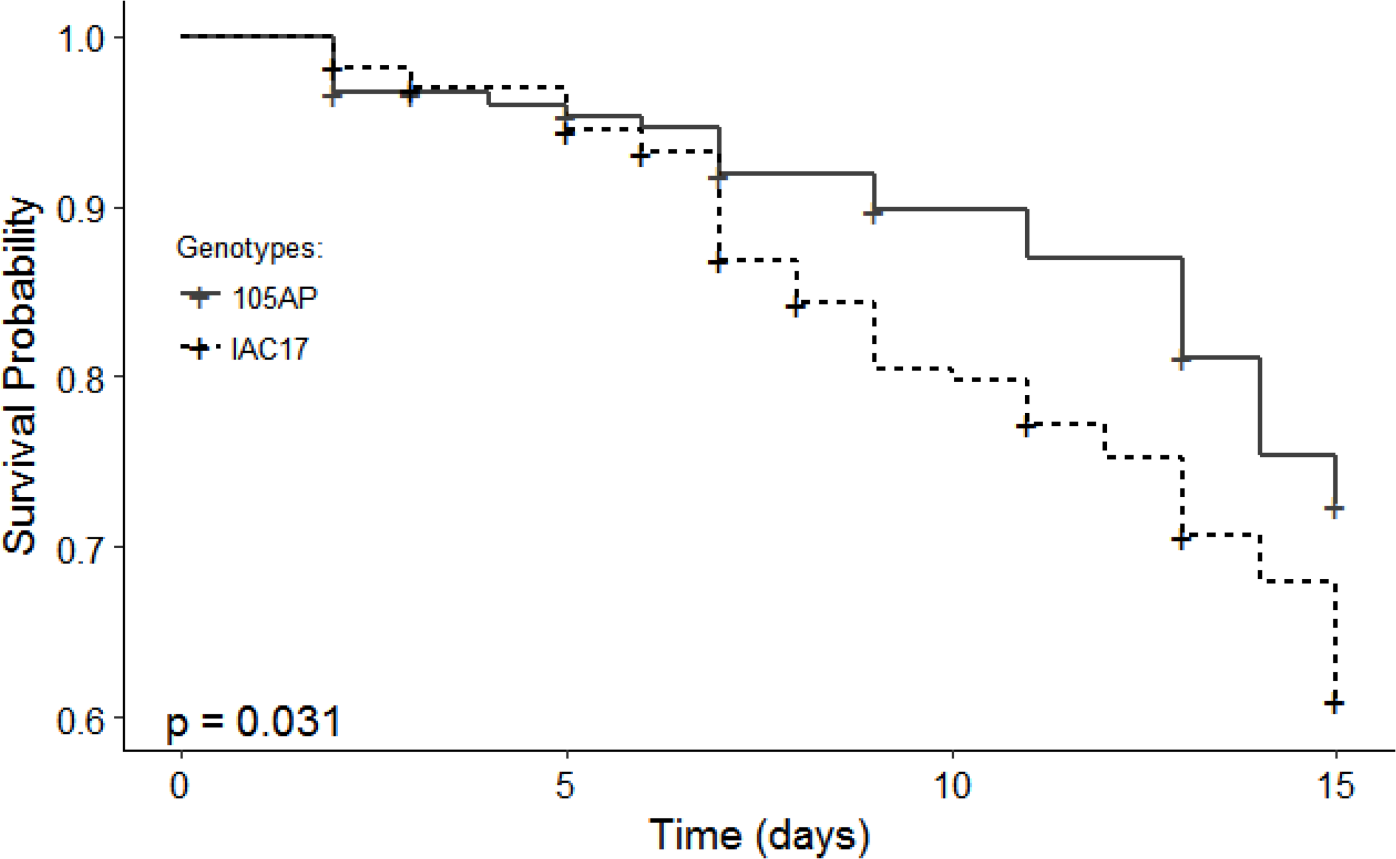
Survival curves of *A*. *gemmatalis* caterpillars fed with leaves from soybean genotypes 105AP and IAC 17, estimated using the Kaplan-Meier method and evaluated statistically by the Log-Rank test.

The higher mortality rate (61% of survival) was observed between 13 and 14 days when for caterpillars fed with IAC 17 genotypes (Fig 2). When comparing the survival curves with the Log-Rank test, a significant difference between the two treatments (*p≤0*.*05*) (Fig 2) was observed. This result agrees with what is already described for these soybean genotypes [3,21], thus IAC 17 genotype was considered resistant and 105 AP genotype, susceptible to *A*. *gemmatalis* caterpillars.

### Quantification of target compounds in soybean leaves

**Fig 3:**
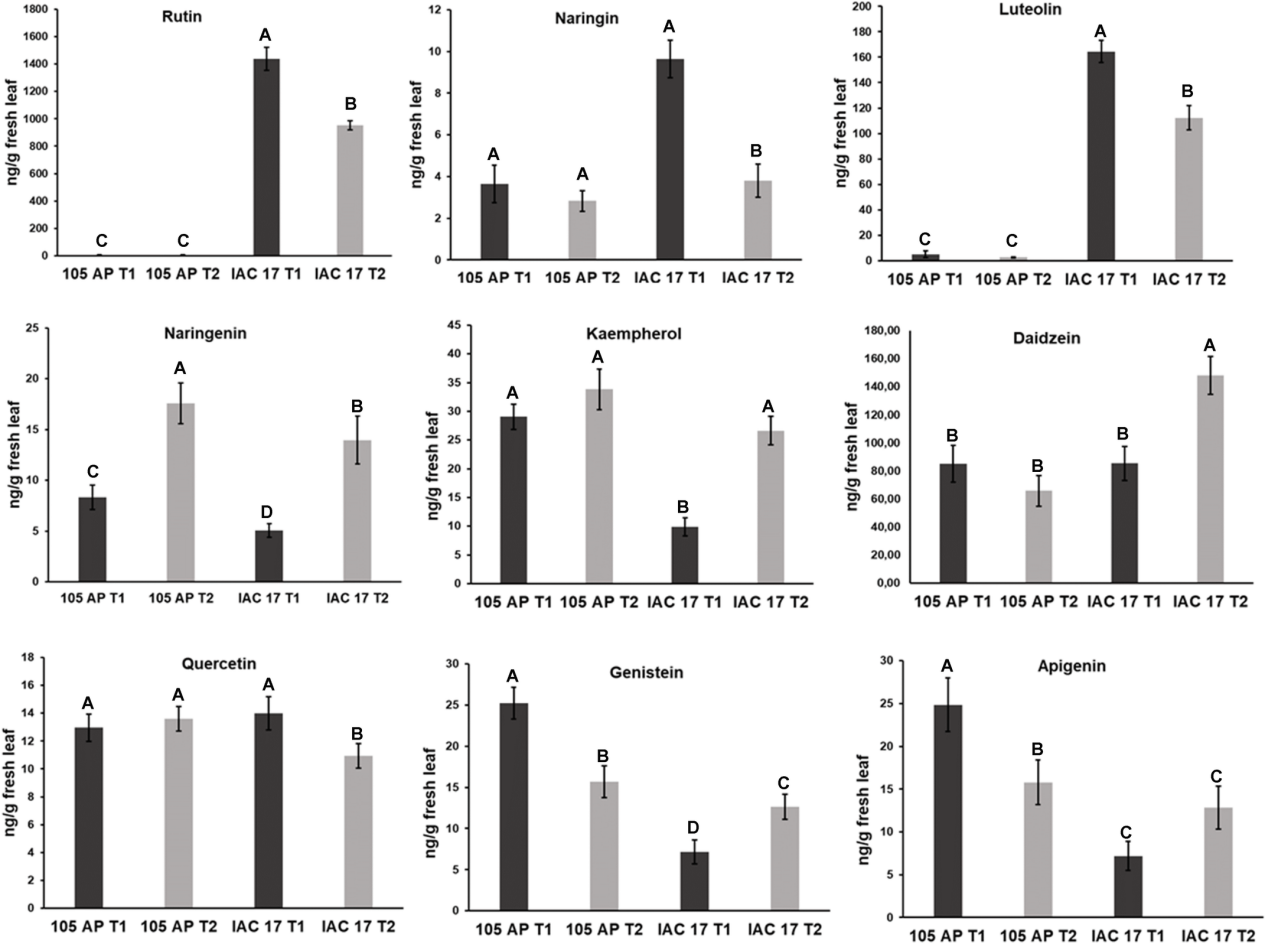
Absolute concentrations of the target flavonoid compounds in soybean leaves from the 105 AP and IAC 17 genotypes, in the presence (T2) or absence (T1) of the *A*. *gemmatalis* caterpillars. Means followed by the same letter do not differ statistically (p < 0.05).

From the 21 standard compounds (S1 Table) analyzed by LC/MS-based target method (Fig 1), some showed mass spectrum and chromatographic signals sufficient to perform the quantitative analysis (Fig 3), while others were detected in very low concentrations or were not synthetized in soybean leaves. Only the glycoconjugates Rutin and Naringin were observed in higher levels in the resistant genotype IAC 17 when compared to the susceptible genotype 105 AP. For the aglycone flavonoid standards, higher concentrations were observed for Daidzein and Luteolin (Fig 3) in soybean resistant genotype IAC 17, while Genistein, Apigenin and Kaempherol were slightly higher in the susceptible genotype 105 AP. The exposition of the soybean leaves to caterpillar’s damage induced an increased relative concentration of Naringenin, Kaempferol, Daidzein, Genistein and Apigenin, especially in the resistant genotype IAC 17 (Fig 3). In contrast, for Rutin, Naringin and Luteolin reductions in their concentrations were observed for IAC 17.

### Profiling of the non-target flavonoids in soybean leaves

**Fig 4:**
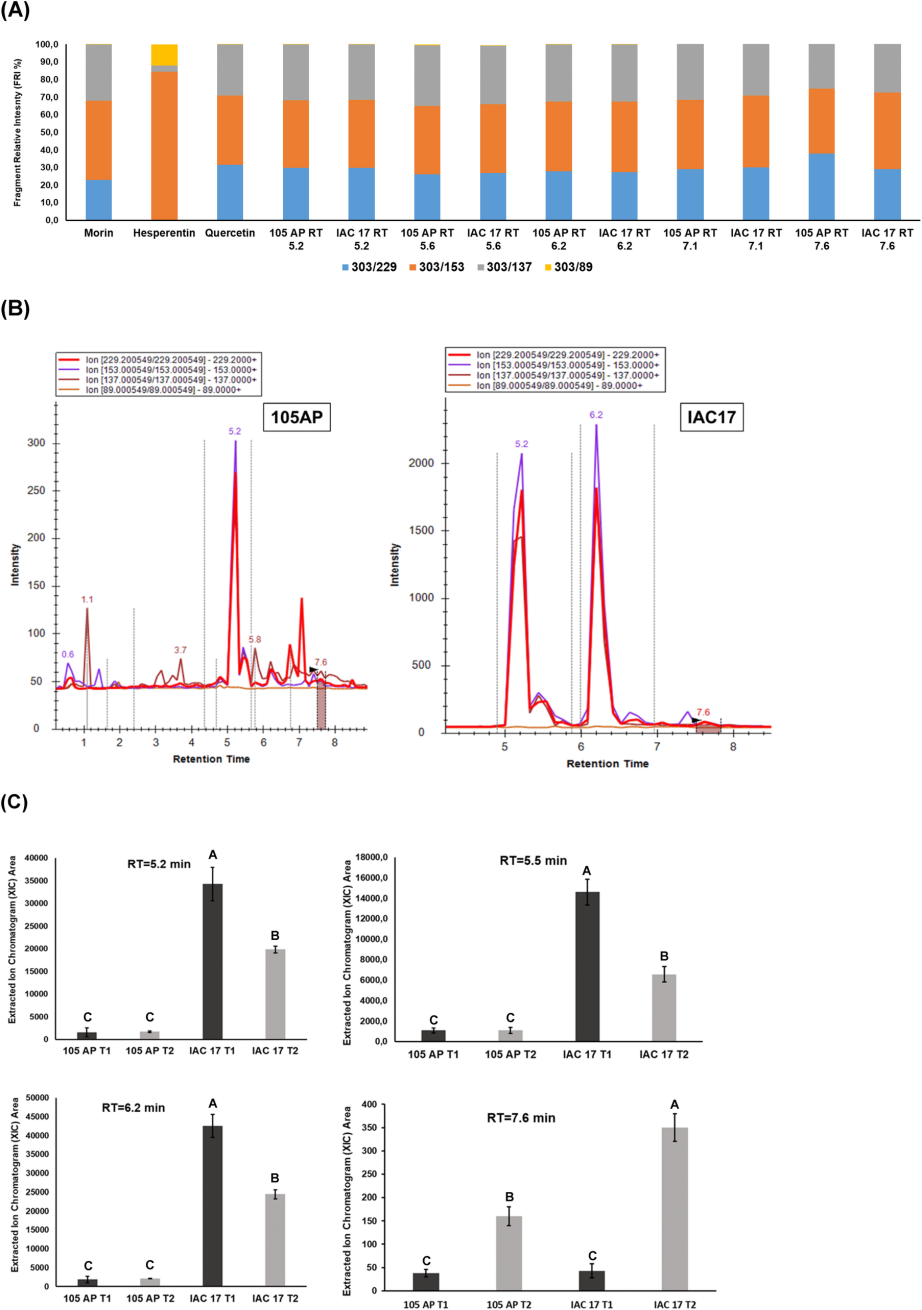
Nontarget analysis of flavonoids from Morin-Hesperentin-Quercetin classes in soybean leaves from the 105 AP and IAC 17 genotypes in the presence or absence of *A*. *gemmatalis* caterpillars. In **(A)**, compounds detected for each genotype, the retention times (RT) observed and their RFI% calculated as in **Fig 1**and **S1 Fig**. The compounds that share the same RFI% patterns related to standard were considered as belonging to a glycoconjugate for those classes. In **(B)** the XICs and the RT of the compounds present in the extracts of soybean genotypes. In **(C)** the relative abundances of some flavonoid compounds characterized as Quercetin conjugates in **(A)** in the presence or absence of *A*. *gemmatalis* caterpillars. Means followed by the same letter do not differ statistically.

**Fig 5:**
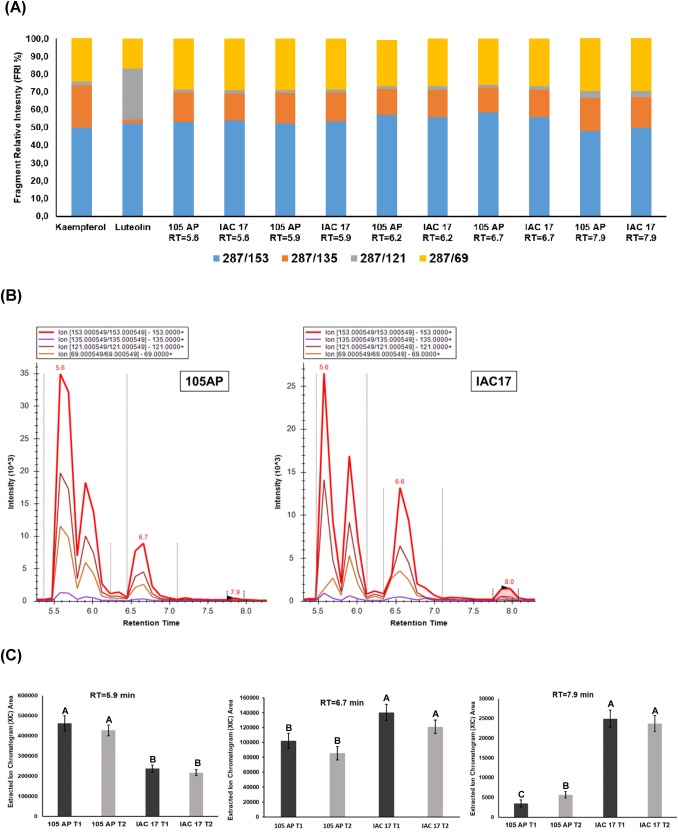
Nontarget analysis of the flavonoids belonging to Kaempferol-Luteolin classes in soybean leaves from the 105 AP and IAC 17 genotypes in the presence or absence of the *A*. *gemmatalis* caterpillars. In **(A),** compounds detected for each genotype, the retention times (RT) observed and their RFI% calculated as in **Fig 1**and **S1 Fig**. The compounds that share the same RFI% patterns related to standard were considered as belonging to a glycoconjugate for those classes. In **(B),** the XICs and the RT of the compounds present in the extracts of soybean genotypes. In **(C),** the relative abundances of some flavonoid compounds characterized as Kaempferol conjugates in **(A)** in the presence or absence of *A*. *gemmatalis* caterpillars. Means followed by the same letter do not differ statistically.

**Fig 6:**
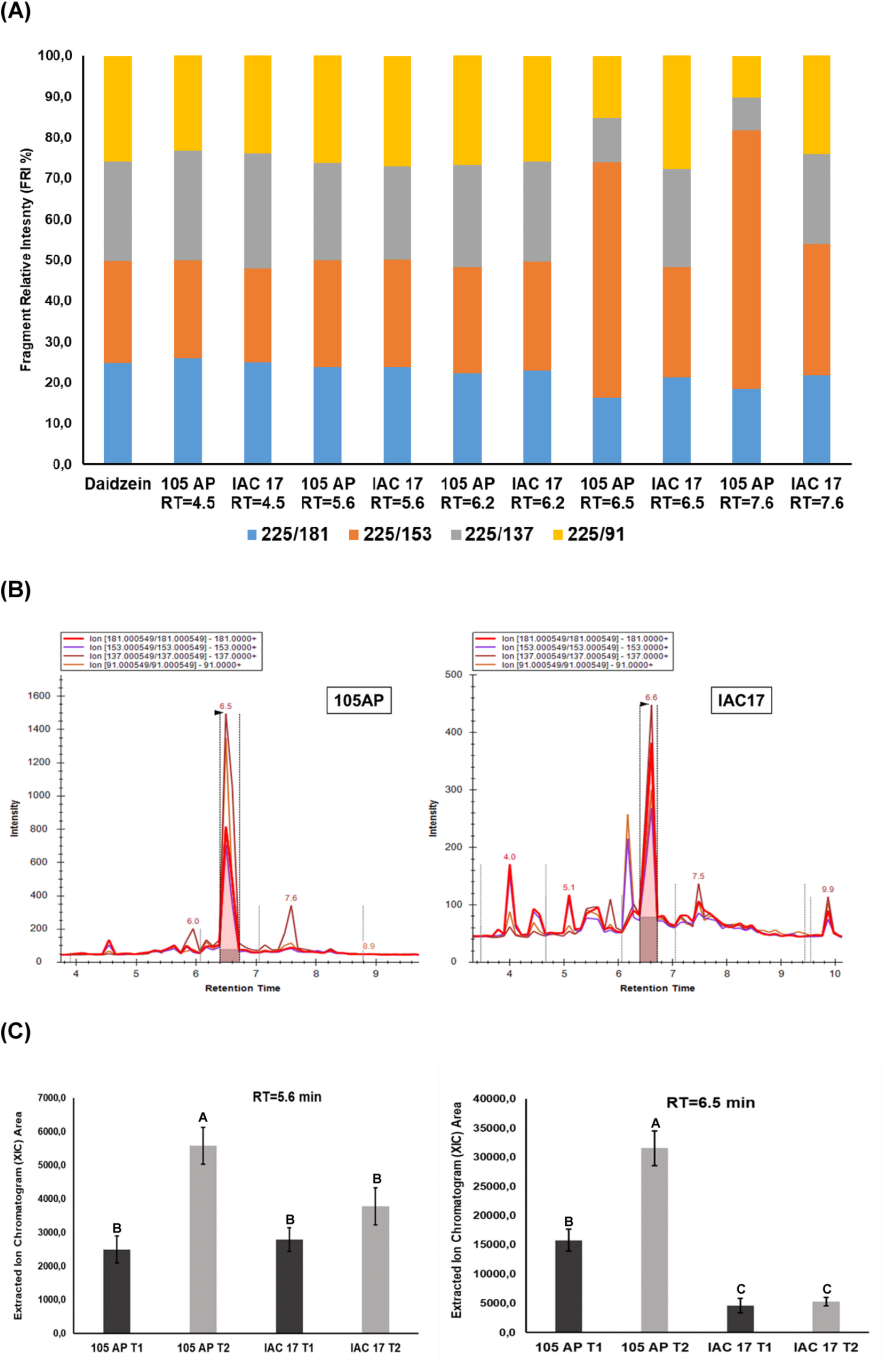
Nontarget analysis of the flavonoids belongings to Daidzein classes in soybean leaves from the 105 AP and IAC 17 genotypes in the presence or absence of the *A*. *gemmatalis* caterpillars. In **(A),** compounds detected for each genotype, the retention times (RT) observed and their RFI% calculated as in **Fig 1**and **S1 Fig**. The compounds that share the same RFI% patterns related to standard were considered as belonging to a glycoconjugate for those classes. In **(B),** the XICs and the RT of the compounds present in the extracts of soybean genotypes. In **(C)** the relative abundances of some flavonoid compounds characterized as Daidzein conjugates in **(A)** in the presence or absence of *A*. *gemmatalis* caterpillars. Means followed by the same letter do not differ statistically.

**Fig 7:**
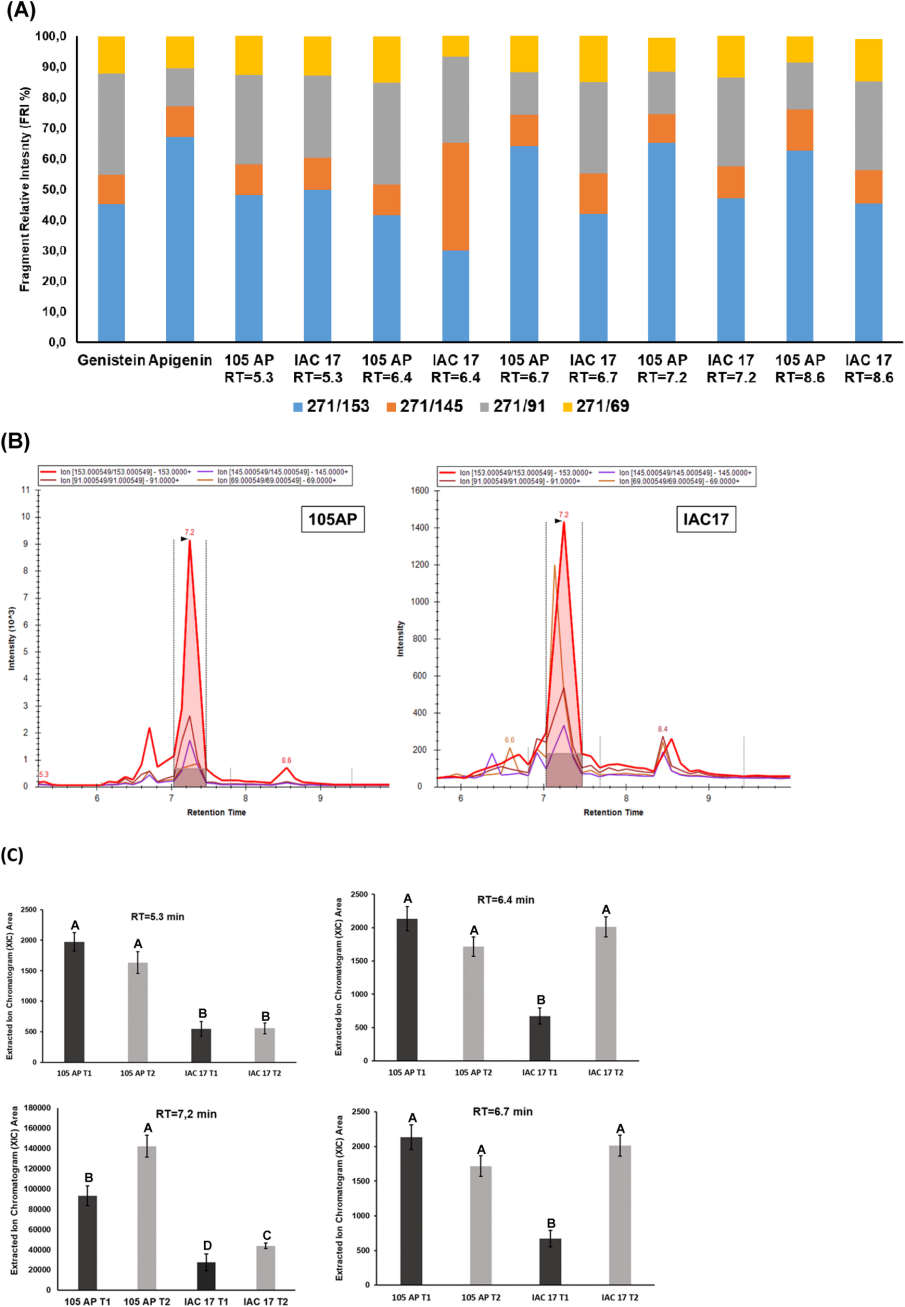
Nontarget analysis of flavonoids belongings to Genistein-Apigenin classes in soybean leaves from the 105 AP and IAC 17 genotypes in the presence or absence of *A*. *gemmatalis* caterpillars. In **(A),** compounds detected for each genotype, the retention times (RT) observed and their RFI% calculated as in **Fig 1**and **S1 Fig**. The compounds that share the same RFI% patterns related to standard were considered as belonging to a glycoconjugate for those classes. In **(B)** the XICs and the RT of the compounds present in the extracts of soybean genotypes. In **(C)** the relative abundances of some flavonoid compounds characterized as Genistein or Apigenin conjugates in **(A)** the in presence or absence of *A*. *gemmatalis* caterpillars. Means followed by the same letter do not differ statistically.

Each commercial standard flavonoid was injected in LC/MS QqQ to generate a pseudo MS^3^ spectrum of the aglycone cores (S2 Table). The fragment relative intensities (FRI%) between the ions were evaluated manually to determinate which m/z values are distinguible between the aglycone isomers (Figs 4A–7A and S3 Table). For example, for Morin the pseudo-MS3/MRM produced the fragment relative intensities (FRI%) of the 22.9% (303>229), 45.1% (303>153), 32.0% (303>137) and 0.1% (303>89). However, for Hesperetin were produced 31.2% (303>229), 39.7% (303>153), 28.8% (303>137) and 0.2% (303>89) and for Quercetin were produced 31.6% (303>229), 39.2% (303>153), 29.0% (303>137) and 0.1% (303>89). These fragmentation patterns allowed to identify the aglycone from the glycoconjugates eluted at the RT = 5.2, RT = 5.6, RT = 6.2, RT = 7.1 and RT = 7.6 min (Fig 4B) as conjugate of the Morin or Quercetin, because the RFI% were highly similar (Fig 4A). The RT = 6.2 min was the same observed for the commercial standard of the glyconjugate Rutin (Fig 3 and S1 Table). However, the glyconjugate for RT = 5.2 min and RT = 5.6 min were only detect by pseudoMS3/MRM method and observed in higher abundance for the resistance genotype IAC 17 (Fig 4B and 4C). Applying the same approach for others flavonoid classes, new compounds showing different retention times were detected. Five XICs with RT of 5.6, 5.9, 6.2, 6.7 and 7.9 min showed RFI% similar to aglycone Kaempferol (Fig 5A) and none for Luteolin. These Kaempferol glycoconjugates were observed with high intensity in soybean leaves, however the relative abundances were similar between genotypes (Fig 5B and 5C). Some XICs generated for this class showed slightly higher abundance (over 10^3^) in the susceptible genotype 105 AP (Fig 5B and 5C), except for RT = 6.7 and 7.9 min which were higher in IAC 17 (Fig 5C). For Daidzein, we observed a RFI% matching with the XICs at RT = 4.5, 5.6 and 6.2 min for both genotypes (Fig 6A and 6B). However, for the XIC at RT = 6.5 and 7.6 min the RFI% matched only for the ions from IAC 17 genotype. Likewise as observed for Kaempferol class, some signals of the XICs detected for Daidzein class shown higher abundance in the susceptible genotype 105 AP (Fig 6B, RT = 6.5 min). However, the profile was more complex for the IAC 17 showing several XICs in different retention times (Fig 6B). The RFI% for RT = 5.6 was similar for both genotypes which is an indicative that this might be the same compound (Fig 6A) and their relative abundances were slightly similar for both genotypes (Fig 6C). The RFI% for the RT = 6.5 min was different for each genotype (Fig 6A) and the abundances were higher in the susceptible 105 AP genotype (Fig 6B and 6C). Thus, for the RT = 6.5 for example, the direct interpretation of the XIC signals without consider the RFI% matches could be erroneous, because the XIC observed can not be the same molecule or flavonoid, albeit have been eluted from LC column at the same RT. In this case, is correct to indicate that this glycoconjugate from Daidzein eluted at RT = 6.5 min was detect only in the resistance genotype.

When applying the pseudo-MS3/MRM method to detect glyconjugates for Genistein and Apigenin, five XICs with high intensities were observed (Fig 7A and 7B). The XICs of the RT = 5.3 min was characterized as a Genistein for both genotypes, while for the RT of 6.7, 7.2 and 8.6 min as Apigenin only from 105 AP genotype, while Genistein were only detected in IAC 17 genotype (Fig 7A and 7B). The XIC of the RT = 6.4 min showed RFI% for Genistein for 105 AP genotype (Fig 7A). In general, the compound abundances were higher for 105 AP genotype (Fig 7C), however the XICs for the RT = 6.7, 7.2 and 8.6 min could not be compared directly, because the fragment used for quantitative analysis showed different intensities for Agigenin and Genistein, as indicate in Fig 7A. Thus, each genotype has high relative content for each specific flavonoid (Fig 7C).

For Myricetin, Phloretin, Catechin and Naringenin classes it was not possible to apply the pseudo-MS3/MRM method, because the mass spectrometry signals were very low (data not showed).

### Determination of the glyconjugate compounds by LC/MS *precursor ion scan* analysis

The pseudoMS3/MRM method only provides structural information about the aglycone core. Therefore, a LC/MS *precursor ion scan* method was applied to determinate which mass molecules were present in the soybean leaves that generated the fragmentation patterns used to characterize the flavonoid classes (item 3.3). The m/z values observed for each XIC (S1 Fig) were search in the mass spectra database *Mass Bank* (http://www.massbank.jp) or in the literature to verify which flavonoids share the same mass observed in the *precursor ion scan*. When *the precursor ion scan* was applied to Morin-Hesperetin-Quercetin class (m/z 303 for aglycone), were observed the precursors m/z = 773.6/RT = 5.2 and m/z = 773.6/RT = 6.2 (S1A, S1B and S1C Fig) with high intensity (10^4^) for the IAC 17 genotype (Fig 4A and 4B) and which the database search Table 1 return as result for the Hesperidin or Rutin (mass of 610,1 Da) (Fig 4B). It is in accordance with the result observed in the standard analysis (Fig 3) and in the class analysis (Fig 4), for the aglycone interpretation from the XIC RT = 6.2 min, as a Quercetin glycoconjugate (Rutin is a Quercetin-3-O-rutinoside).

**Table 1:** Precursor ions detected by *precursor ion scan* method and the identified flavonoid glycoconjugates.

Flavonoid Class	Precursor m/z	RT (min)	Conjugate (m/z)	Relative Abundance in XIC (%)	Sugar Moiety (mass)	FRI% Match	Mass Bank Database/Literature
Daidzein	255	6.5	503.3	100	248.3	+/-	Daidzein 7-O-glucoside-O-6-malonate^1^
	255	7.6	700.1	100	445.1	+/-	NH
	255	7.6	647.7	55	392.7	+/-	NH
	255	6.2	503.3	100	248.3	+	Daidzein 7-O-galactoside-O-6-malonate ^1^
	255	6.2	581.7	37.35	326.7	+/-	NH
	255	4.5	417.3	100	162.3	+	Daidzein-7-O-glucoside^1^
	255	4.5	452.0	61.94	197	-	NH
	255	5.6	757.1	100	502.1	+	NH
	255	5.6	417.1	85.88	162.1	+	Daidzein-7-O-glucoside^1^
	255	5.6	549.4	47.53	294.4	-	NH
Genistein-Apigenin	271	7.2	519.0	100	248	105AP IAC17	Apigenin 7-O-6-O-malonylglucoside ^3^ Genistein 7-O-6-O-malonylglucoside ^1^^,^ ^3^
	271	6.7	433.0	100	162	105AP	Apigenin-7-O-glucoside ^4^
	271	6.7	565.3	18.23	294.3	105AP	Apigenin 6—xyloside-8—glucoside ^4^
	271	6.7	767.3	14.2	496.3	IAC17	Genistein O-diglucoside dimalonylated ^4^
	271	6.4	565.8	100	294.8	+	Genistein 6-C-xyloside-8-C-glucoside ^4^
	271	6.4	595.2	42.4	322.2	+	Genistein 4,0-7-diglucoside ^3^
	271	5.3	594.8	100	323.8	+	NH
	271	8.6	519.3	100	248.3	105AP IAC17	Apigenin 7-O-6-O-malonylglucoside ^3^ Genistein 7-O-6-O-malonylglucoside ^3^
Luteolin- Kaempherol	287	5.6	757.4	100	470.4	+	Kaempferol-3-O-glucopyranosyl-O-rhamnopyranosyl-galactopyranoside ^7^^,^ ^8^
	287	5.9	611.4	100	324.4	+	Kaempferol-3-O-di-galactopyranoside ^7^^,^ ^8^
	287	5.9	741.4	41.67	454.4	+	Kaempferol-3-O-di-rhamnopyranosyl)-galactopyranoside ^7^^,^ ^8^
	287	5.9	595.4	28.33	308.4	+	Kaempferol-3-O-rhamnopyranosyl- galactopyranoside ^7^^,^ ^8^
	287	6.7	595.3	100	308.3	+	Kaempferol-3-O -rhamnopyranosyl- glucopyranoside ^7^^,^ ^8^
	287	6.2	785.4	100	498.4	+	NH
	287	6.2	611.2	97	324.2	+	Kaempferol 3-O-beta-diglucoside ^6^^,^ ^7^
	287	6.2	757.4	68	454.4	+	Kaempferol-3-O-glucopyranosyl-rhamnopyranosyl-glucopyranoside ^5^^,^ ^7^
	287	7.9	967.0	100	680.8	+	Kaempferol-3-feruloyl-di-glucoside-7-glucoside ^6^
Morin-Hesperentin-Quercetin	303	5.2	773.6	100	470.6	+	Quercetin 3-*O*-rhamnosyl-glycoside-7-*O*-glucoside ^2^
	303	5.5	627.5	100	324.5	+	Quercetin-di-glucoside ^2^^,^^3^
	303	5.5	757.3	71.59	454.3	+	Quercetin-3-O-rhamnopyranosyl- glucopyranoside-rhamnopyranoside ^2^ ^,^^4^
	303	7.1	465	100	162	+	Isoquercetin ^4^^,^ ^6^
	303	6.2	611.2	100	308.2	+	Rutin ^4^^,^ ^6^
	303	7.6	647,5	100	444.5	+	NH

^**1**^ describe by Wu et al., 2004

^**2**^ Lin et al., 2008

^**3**^ Boue et al., 2003

^**4**^ MassBank

^5^ Zhou et al., 2014

^6^ Olsen et al 2012

^7^ Ho et al, 2002

^8^ Song et al., 2014.

**+:** when the FRI% match for both genotype. **+/-**: when the **FRI%** match only for one genotype. **NH**: no hit in database or literature. **Relative Abundance in the XIC (%):** percentage of the relative intensity in mass spectrum as showed in the **S1 Fig**.

Also, glyconjugates for Daidzein, Daidzein-7-O-glucoside (m/z 417.3 for the RT = 4.5 and 5.6 min) and Daidzein 7-O-glucoside-O-6-malonate (m/z = 503.3 and RT = 6.2 and 6.5 min) were observed. The same m/z values eluted at different retention times is an indicative of the presence of isomers for the same Daidzein glycoconjugate (Table 1) or containing different sugar moieties. For some m/z values, it was not possible to identify in the database or in the available literature, flavonoids that share the same nominal mass (Table 1). In addition, for some XICs more than one ion was observed. These m/z values and their relative intensities are indicated in the Table 1. For example, for Daidzein class, ions m/z 756.1, 417.1 and 549.4 were detected at RT = 5.6 min. These data is an indicative that different molecules were co-eluted from analytical column. Others glycoconjugate flavonoids that were also identified in the database or literature belonging the classes Genistein-Apigenin and Luteolin-Kaempferol are also indicated in the Table 1.

### Global analysis of the flavonoid profiles

**Fig 8:**
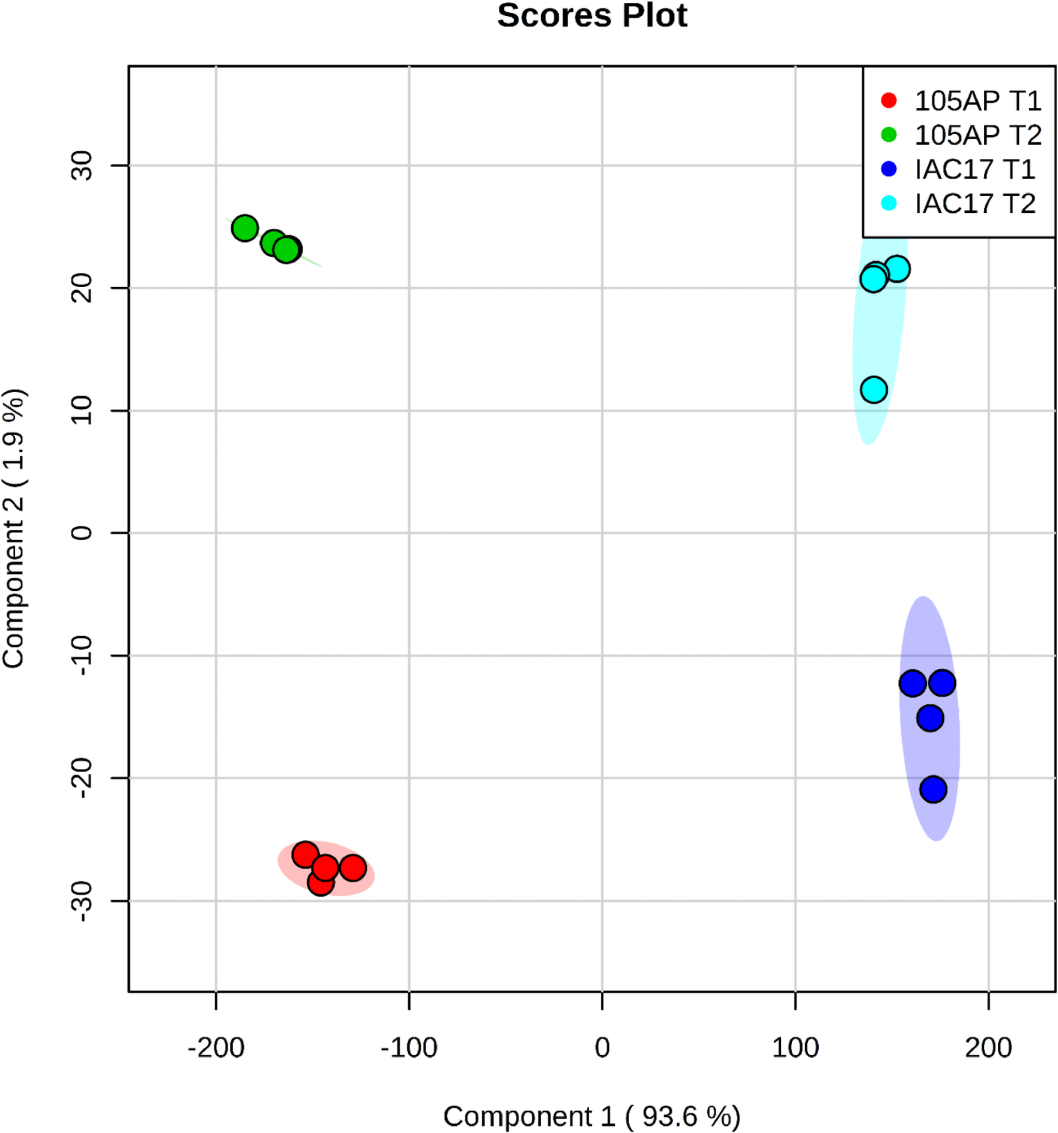
Clustering analysis by *PLSDA* method of the characterized flavonoids in soybean leaves from the 105 AP and IAC 17 genotypes in the presence or absence of the *A*. *gemmatalis* caterpillars.

The PLSA-DA method was used to analyze the samples (S4 Table) according to the most significant variables. It was observed a differential grouping for the contrasting genotypes and caterpillar treatment (Fig 8). As can be observed in Fig 8, the experimental variance was separated in two distinct groups, which mainly reflect the genotypes differences. The first principal component (PCA 1) explained the greatest variance (93%) across the data and separated the samples based on genotypes. The second principal component (PCA2) also separated the data but based in the treatments, however with a lower variance (1.9%). It was attributed to a reduced effect of the treatment with caterpillar over the abundance of characterized compounds (Fig 8).

**Fig 9:**
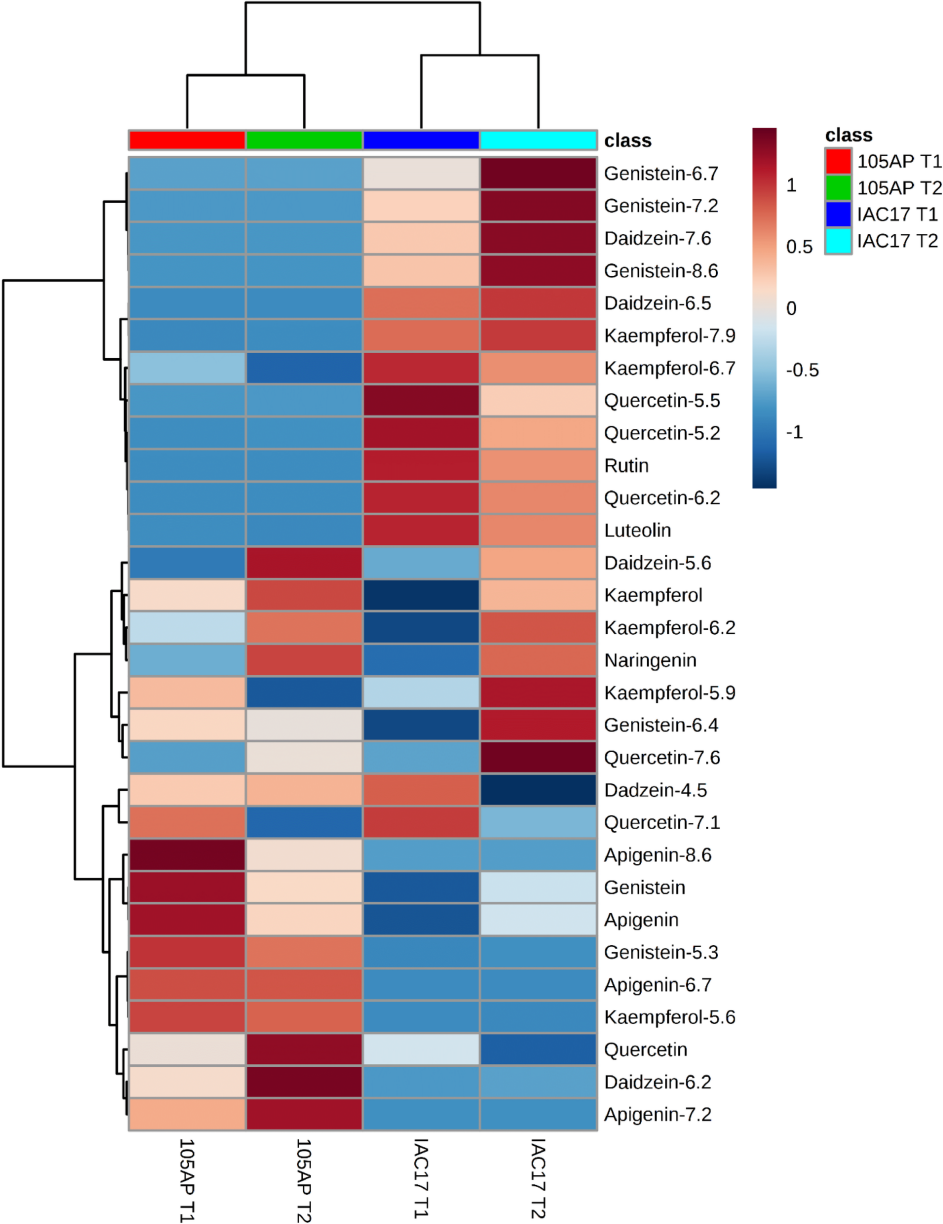
Clustering analysis by HeatMap method of the characterized flavonoids in soybean leaves from the 105 AP and IAC 17 genotypes in the presence or absence of the *A*. *gemmatalis* caterpillars.

These differences could also be observed in the HeatMap analysis, which indicated some compounds in higher abundance in specific genotypes and that this level was increased when caterpillars were present (Fig 9). Cleary, it’s possible to observe that a first cluster was formed by 12 compounds: Genistein-6.7, -7.2 and -8.6; Daidzein-7.6 and -6.5; Kaempferol-7.9 and -6.7; Quercetin-5.2, -5.5 and -6.2 and Luteolin, which showed higher concentrations in the resistant genotype IAC 17. In contrast, 9 compounds formed a second cluster, detected in lower relative concentrations in IAC 17 genotype: Apigenin-8.6, -5.3, -6.7 and -7.2; Apigenin, Genistein and Quercetin aglycones; Kaempferol-5.6; Daidzein-6.2 (Fig 9). In a thirty cluster, compounds that showed increased in similar levels in both genotypes in the presence of caterpillars were grouped.

**Fig 10:**
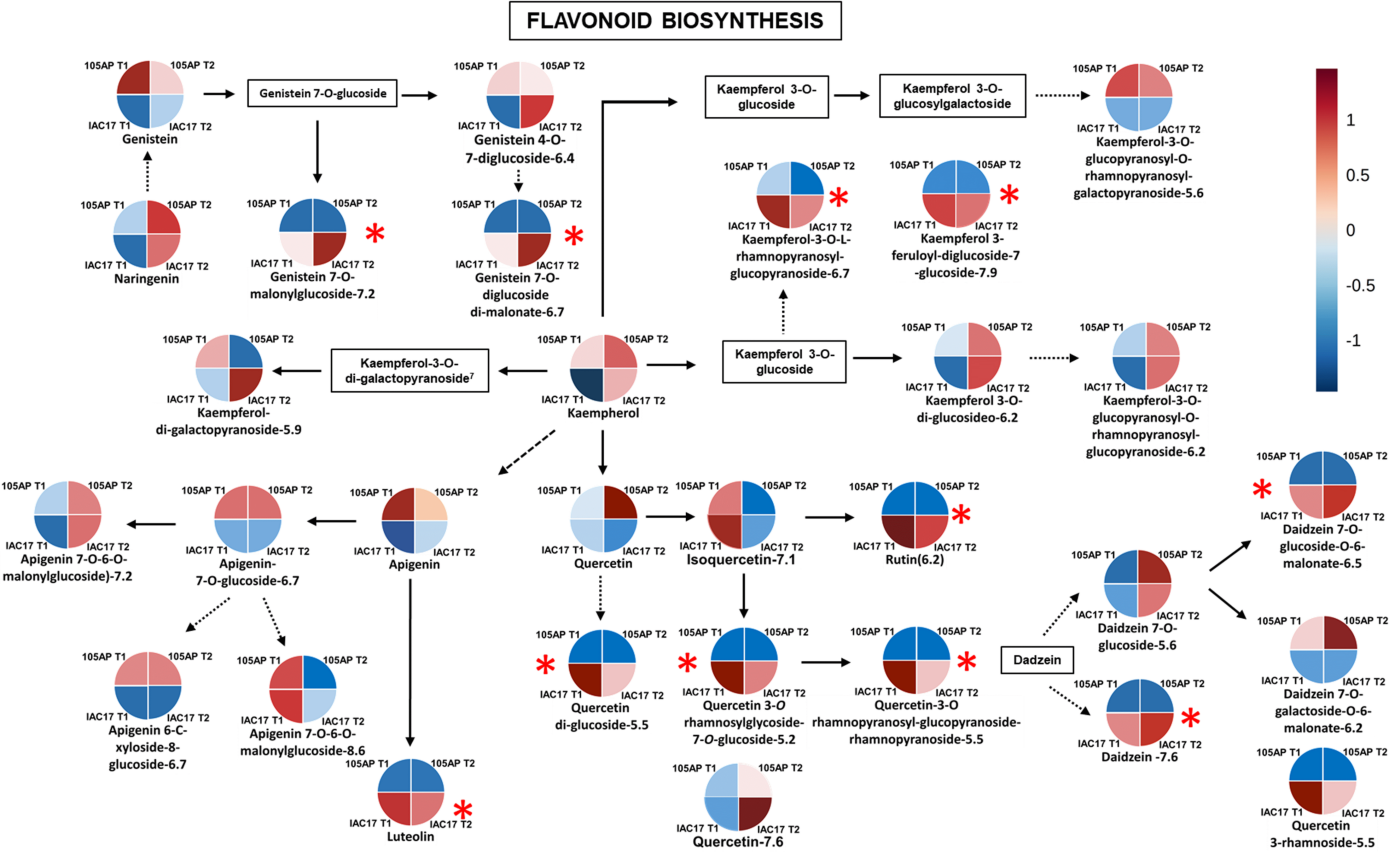
Overview of flavonoid biosynthesis pathway reconstructed using the characterized compounds from soybean leaves from the 105 AP and IAC 17 genotypes, in the presence or absence of *A*. *gemmatalis* caterpillars. Each quadrant of the circle represents each genotype and treatment. The color of each quadrant follow the same pattern from the HeatMap (Fig 9) and is an indicative of the correlation analysis and express the levels of increase and decrease on the abundance of flavonoid for each genotype and treatment. A red asterisk indicates compounds that were more abundant specifically in the resistant genotype IAC 17.

Profiles can also be represented in a metabolic pathway map of flavonoid biosynthesis (Fig 10). Thus, it was possible to observe classes that have been altered by differences in the metabolism of the genotypes and in response to caterpillar damage. Four Quercetin glyconjugates displayed high relative abundances in the resistant IAC 17 genotype, including Rutin and Quercetin 3-*O*-rhamnosylglycoside-7-*O*-glucoside. Likewise, Rutin was observed in the highest concentration at IAC 17 genotype (Fig 3). Thus, this pathway for biosynthesis of the compounds was highly active in this genotype. Pathways for the Glyconjugates of Genistein, Dadzein and Kaempferol were also actived (Fig 10). Some glyconjugates that were identified in the both soybean leaves, were not found described in soybean reference KEEG database, and could be a new flavonoid, such as Kaempferol-3-feruloyl-diglucoside-7-glucoside RT = 7.9) (Fig 10), present in high abundance in the resistant genotype IAC 17 (Fig 10 and Fig 5).

## Discussion

Flavonoids act in a wide range of plant mechanisms, from physiological development to plant responses to abiotic and biotic stresses [5,6,12]. Moreover, due to their health-promoting effects. flavonoids are of pharmaceutical interest. Herbivores damage is a major biotic stress for terrestrial plants. In order to survive to this stress, plants have evolved various defenses including the production of secondary metabolites such as alkaloids, terpenoids, flavonoids, and glucosinolates [12]. Thus, a more detailed metabolic profiling between soybean genotypes is important for a more comprehensive characterization of the resistance to insect damage. However, the study of flavonoid content in plants is challenging, because their chemical complexity and cause traditional methods based in LC/MS are time-consuming [24] and could underestimated the full chemical diversity in plant tissues.

When using a target method based in 21 commercial standards, only three flavonoids were observed; Rutin, Luteolin and Dadzein (Fig 3) in high abundance in soybean leaves from the IAC 17 resistant genotype. When applying the standard-based method, Rutin was characterized (RT = 6.2 min) as the major Quercetin glyconjugate present in higher concentrations in the resistant genotype (Fig 3). However, when applying non-target method, besides Rutin, others five glycoconjugates from Quercetin class in similarly high relative abundance were identified (Fig 4B and 4C), as evidenced at RT = 5.2 min. Thus, target method underestimated the complexity of Quercetin class.

Flavonoid profiles from soybean tissues can variate when applying different traditional LC and LC/MS methods and also due to evaluated standards, as well as, soybean genotypes with different genetic backgrounds [9, 25–27], plant structure and developmental stages [24, 26, 28, 29]. Distinct profiles for 105 AP and IAC 17 genotypes were also observed, revealed by PLSDA analysis (Fig 8). However, the full extension of these variations was only possible to access when the non-target method was applied. Ho et al. (2002) verified that flavonoids in the soybean leaves were mainly Kaempferol glycosides, whereas in the soybean seeds, mainly isoflavone glycosides and derivatives are found. While the Piubelli et al. (2005) verified high Rutin (Quercitin 3-O-rhamnosyl glucoside) and Genistin (genistein 7-O-glucoside) contents in soybean leaves of different genotypes. In the generated profiles, Kaempferol glicoconjugates were detected, but also high abundances for Rutin, some Genistein and Daidzein glycoconjugates were found (Fig 3 and Fig 7). In addition, the signals for the Kaempferol conjugates were higher (10^3^) indicating that these are the most abundant flavonoids in soybean leaves (Fig 5B) from both genotypes. The non-target method showed to be efficient for allowing the detection of several glycoconjugates, as well as for flavonoids analysis directly from metabolic extracts, without purification stages [24]. However, for compounds at very low concentration, the fragments used to generate the RFI% could be absent or with a signal/noise relation that will produce fluctuation at the relative proportion, as evidenced for RT = 7.1 min (Fig 4A and 4B). Thus, the homogeneity and reproducibility of the RFI% need to be verified (S2 Fig).

Distinct profiles between soybean genotypes could also be verified for Genistein and Apigenin classes, which Genistein being more abundant in the resistant genotype IAC 17, and Apigenin more abundant in the sensitive genotype, 105 AP (Fig 7 and Fig 10). The highest distinct flavonoid profiles were also indicated by PLSDA and HeatMap analysis (Fig 8 and Fig 9), with an expressive separation between genotypes (over 95% of variance observed), and a correlation with the survival curve behavior of *A*. *gemmatalis* caterpillars (Fig 2). The mortality was higher for caterpillars fed with IAC 17 genotype when compared to 105 AP genotype, characterized as resistant. However, this correlation cannot be attribute to all evaluated flavonoids, because some compounds were detected in similar or lower abundance in the resistance genotype (Fig 9). Thus, these cluster analysis indicated which compounds could specifically affect the caterpillar survivor (Fig 9 and Fig 10) and these were identified as glycosylate flavonoids belonging to Quercetin, Daidzein, Genistein and Kaempferol classes (Fig 10).

Flavonols such as Rutin and its glycosylated forms have been reported to enhance the mortality rate and inhibit the growth of *Spodoptera litura* (Fabricius, 1775) (Noctuidae: Lepidoptera) caterpillars on groundnut (*Arachis hypogaea* L.), *Arabidopsis* [30, 31] and soybean [3, 9]. Extracts from leaves of resistant soybean genotypes negatively affected the caterpillars’ physiology and behavior of *Heliothis virescens* (Fabricius, 1777) *Trichoplusiani* (Hübner, 1800) (Noctuidae: Lepidoptera) [32, 33] and *A*. *gemmnatalis* [9]. Three isoflavonoids (Coumestrol, Phaseol and Afrormosin) were isolated from methanolic fractions of soybean foliage and caused mortality of *Pseudoplusia includens* [34]. Incorporation of Coumestrol, which was associated with the resistant soybean genotype, into a modified artificial diet resulted in significant reductions in the weight gain for *Pseudoplusia includes* larvae [35]. Soybean extracts added to artificial diets are mainly composed of Rutin, Quercitin 3-O-glucosylgalactoside and genistin (genistein 7-O-glucoside) [9, 30, 31], same compounds observed in the profiles presented here. The non-target method turns possible to detect others derivatives also in high abundances in the resistant genotype (Fig 9 and Fig 10). Beyond those active compounds in the diet, three more Quercetin conjugates were observed: Quercetin 3-rhamnoside, Quercetin 3-*O*-rhamnosylglycoside-7-*O*-glucoside and Quercitin-3-O-rhamnopyranosyl-glucopyranoside-rhamnopyranoside; and two more Genistein conjugates: Genistein-7-O-diglucoside-dimalonylated and Genistein-7-O-6-O-malonyl glucoside (Fig 10); that were detected in high relative abundances in genotype IAC 17 (Fig 4). The non-target method allowed broader comparisons between contrasting genotypes, because all compounds belonging to flavonoid classes and present in high concentrations were included. This subtractive approach indicated possible candidate compounds for *in vitro* biological activity assays, especially those compounds present in high relative concentrations. Thus, the non-target method is more appropriate for a broad range profiling when contrasting genotypes are evaluated and when the objective is to correlate these profiles to biological activities.

The flavonoids are phenolic compounds with addition of sugar moiety that differ mainly at the position and conformation of the chemical groups. Although ions intensity from different molecules are not directly comparable, these compounds will have similar proton affinities. Thus, the XIC signals from different flavonoid classes (for example for Quercetin, Genistein, Kaempferol and Daidzein) could reflect the relative concentration in soybean leaves. Taking this into account, the most abundant flavonoid in soybean leaves belonged to the Kaempferol and Quercetin classes. However, only the Kaempferol-3-O-L-rhamnopyranosyl-glucopyranoside and Kaempferol-3-feruloyl-diglucoside-7-glucoside presented higher levels in the resistant genotype IAC 17. In contrast, four Quercetin glycosylates were higher in the IAC 17 genotype (Figs 8 and 9) and their derivatives could be responsible for caterpillar inhibition, as observed *in vitro* and *in vivo* assays [3, 9, 11, 36, 37]

In the metabolic pathways analysis (Fig 10), the biosynthetic pathway for Quercetin derivatives was active just in the resistant genotype, albeit their relative abundances slightly decreased in the presence caterpillar damage (Fig 4 and Fig 10). Beetle damage induced in soybean leaves, significantly increased in the concentrations of Naringenin methyl hexose, Kaempferol diglycoside, Kaempferol triglycoside, and Quercetin triglycoside [26]. However, it was slightly 30% more abundant in the elicited soybean foliage. In soybean resistance genotypes infested with *Piezodorus guildinii* (Westwood, 1837) (Pentatomidae: Hemiptera) concentrations of Rutin and Genistein flavonoids showed a little increased [38].

For some flavonoids that showed high relative abundance in the IAC 17 genotype, it was observed decreased in the concentration after caterpillar damage (Figs 8 and 9). Thus, can be suggested that caterpillar damage did not elicit the plant defense responses and that resistance properties are apparently a constitutive characteristic of this genotype. The synthesis decrease could be also justify by metabolic adjustment induce by phytohormones like jasmonic acid. In another way, biosynthesis of some flavonoids (Daidzein-7-O-glucoside, Genistein-4,0-7-diglucoside, Kaempferol-3-O-rhamnopyranosyl-glucopyranoside, Kaempferol-3-O-beta-di-glucosideo, Kaempferol-3-O-glucopyranosyl-rhamnopyranosyl-glucopyranoside, Kaempferol-3-O-di-galactopyranoside, Kaempferol-3-O-di-O-rhamnopyranosyl-galactopyranoside, Kaempferol-3-O-rhamnopyranosyl-galactopyranoside) were significantly increased by caterpillar damage (Fig 9 and Fig 10).

*Spodoptera litura* damages on soybean leaves displayed an induced accumulation of flavone and isoflavone aglycones 4’,7-dihyroxyflavone, daidzein, formononetin, and the isoflavone glucoside daidzin [27]. It was also observed that a reduction in kaempferol-3,7-dirhamnoside (KRR) corresponded to an increased susceptibility of *Arabidopsis* plants to *Pieris brassicae* (Linnaeus, 1758) (Pieridae: Lepidoptera) caterpillars which also had their grow affected when fed with artificial diet containing KRR [10]. The function of this compound is supported by a direct defense against this specialist caterpillar. Thus, the complex profile of the Kaempferol glyconjugate observed in soybean leaves (Fig 10) could be a response to a specialist herbivory as *A*. *gemmatalis*. The effect of some flavonoid glyconjugates included in the caterpillars diet have been evaluated, however these compounds belong mainly the quercetin classes. Some reports indicated also that kaempferol glycoconjugates could also be important for inhibit *Pieris brassicae* caterpillars [10].

The fine regulation of flavonoid biosynthesis is achieved by a combinatorial action of transcription factors, belonging to different transcription factor families, involved in the transcriptional control of flavonoid biosynthesis genes [10, 12, 39]. Stracke et al. (2010) demonstrated a differential influence of the transcriptional factors MYB11, MYB12 and MYB111 on the spatial accumulation of specific flavonol derivatives in *Arabidopsis* leaves, such as Quercetin 3-O-rhamnoside, Quercetin 3-O-rhamnoside-7-O-glucoside, Quercetin 3-O-rhamnosyl glucoside-7-O-rhamnoside. In accordance, in the present profiles the difference of genetic background between genotypes and the elicitation by the caterpillar damage culminate in alterations on the relative abundance of Quercetin/Kaempferol conjugates (Fig 10). Onkokensung et al. (2014) also showed in *Arabidopsis* that an enhance in the activity of anthocyanin pathway results in alterations of Quercetin/Kaempferol derivatives, which has a negative effect on the accumulation of Kaempferol-3,7-dirhamnoside, a novel defensive metabolite against a specialist caterpillar.

## Concluding remarks

Considering *A*. *gemmatalis* survival percentages when fed with leaves from soybean IAC17 genotype, is was possible to characterized that this genotypes might present an antibiosis-type resistance, and that UFV 105 AP genotype is more suitable for insect development. Applying a combination of target and non-target methods LC/MS-based, several flavonoids were characterized from soybean leaves. However, the non-target method showed a broad range of compounds in the profile and was efficient to characterize the metabolic response pathway involving flavonoids from contrasting genotypes to resistance to *A*. *gemmatalis*. Some flavonoid glyconjugates for Quercetin, Genistein, Keampferol and Dadzein showed higher concentrations in soybean resistant genotype, being Kaempferol and Quercetin the most abundants. Nevertheless, just one Kaempferol conjugate was higher in the resistant genotype IAC 17, in contrast to three glycosylated Quercetin. Metabolic pathways analysis elucidated the biosynthetic pathway for Quercetin derivatives and showed to be more active in the resistant genotype. In another way, the susceptible cultivar 105 AP showed higher abundance of Kaempferol-based flavonoids. It can be an indicative of the genetic background for plant defenses against caterpillar damages related to biosynthetic pathway activity for secondary compounds, eliminated after successive crosses. The broad range overview of the profiles generated by the non-target method could be used as basis for directing the genetic studies related to regulatory mechanisms of these pathways, as well as to support breeding programs for herbivory resistance in soybean.

## Supporting information

S1 Fig Precursor ion scan results used to determinate the glycoconjugate mass. In (**A**) the total ion chromatogram (TIC) and in (**B**) and (**C**) the mass spectrum of the precursor ion.
(DOCX)

S2 Fig Skyline analysis of the flavonoid classes by LC/MS. (DOCX)

S1 Table Transition list used as input for skyline analysis of the commercial phenolic compound used as standards. (DOCX)

S2 Table Transition list used as input for Skyline in the flavonoid class analysis. (DOCX)

S3 Table Transition list used as input in the Skyline analysis for the profiling of the flavonoid compounds in the soybean leaf. (DOCX)

S4 Table Flavonoid intensities for each genotypes and treatment used as input in the MetaboAnalyst platform. (DOCX)

S5 Table Absolute and relative abundances of the all compounds identified by LC/MS approaches. The data were used for statistical and clustering analysis using the *MetaboAnalyst* platform.
(DOCX)
