# Supplementary figures and images for: Broad range flavonoid profiling by LC/MS of soybean genotypes contrasting for resistance to Anticarsia gemmatalis (Lepidoptera: Noctuidae)
Source: PLoS One. 2018 Oct 3;13(10):e0205010. doi: 10.1371/journal.pone.0205010 (PMC6169965; doi:10.1371/journal.pone.0205010)

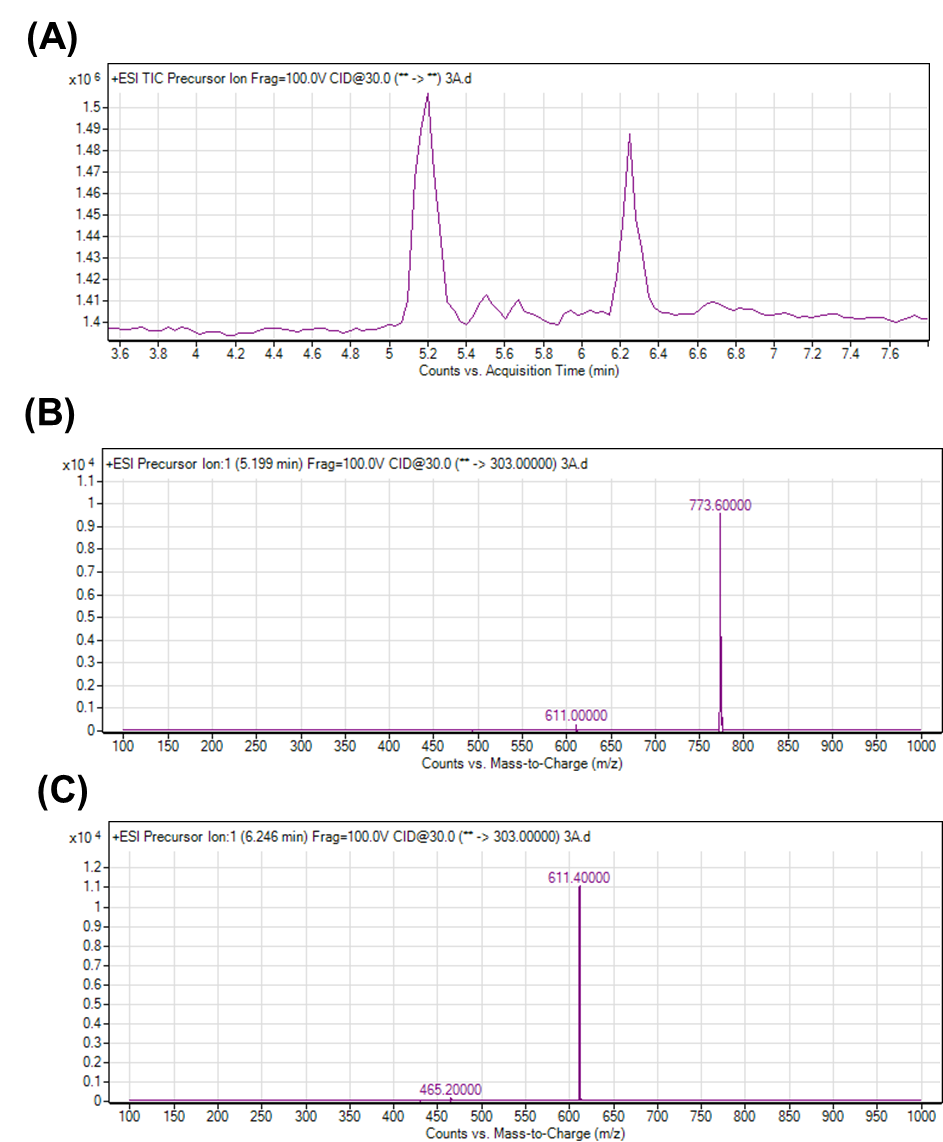

Supplement: S1 Fig — In (A) the total ion chromatogram (TIC) and in (B) and (C) the mass spectrum of the precursor ion. (DOCX) [file pone.0205010.s001.docx]

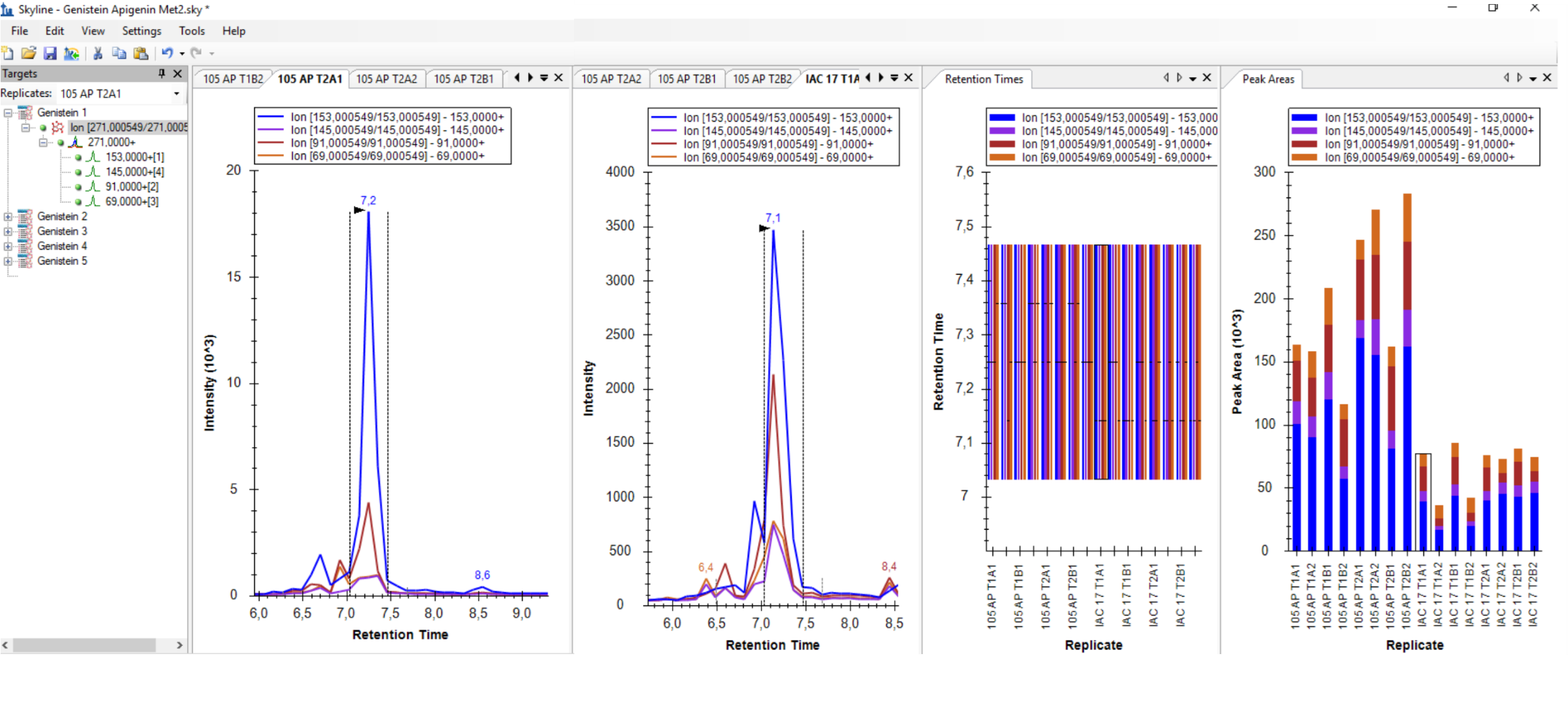

Supplement: S2 Fig — (DOCX) [file pone.0205010.s002.docx]
