# Supplementary material for: Broad range flavonoid profiling by LC/MS of soybean genotypes contrasting for resistance to Anticarsia gemmatalis (Lepidoptera: Noctuidae)
Source: PLoS One. 2018 Oct 3;13(10):e0205010. doi: 10.1371/journal.pone.0205010 (PMC6169965; doi:10.1371/journal.pone.0205010)
Supplement: S1 Table — (DOCX) [file pone.0205010.s003.docx]

**Table S1**: Transition list used as input for skyline analyzes of the commercial phenolic compound used as standard.

| **Molecule Name** | **Precursor Charge** | **Product**  **m/z** | **Product charge** | **Precursor RT** | **Precursor CE** | **Precursor m/z** | **Polarity** |
| --- | --- | --- | --- | --- | --- | --- | --- |
| **Hesperidin** | 1 | 303 | 1 | 6.8 | 30 | 611 | Positive |
| **Rutin** | 1 | 303 | 1 | 6.2 | 30 | 611 | Positive |
| **Naringin** | 1 | 273 | 1 | 6.8 | 30 | 581 | Positive |
| **Orientin** | 1 | 329 | 1 | 5.8 | 30 | 449 | Positive |
| **Isoorientin** | 1 | 299 | 1 | 5.6 | 30 | 449 | Positive |
| **Vitexin** | 1 | 313 | 1 | 6.2 | 30 | 433 | Positive |
| **Isovitexin** | 1 | 283 | 1 | 6.2 | 30 | 433 | Positive |
| **Myricetin** | 1 | 153 | 1 | 7.2 | 30 | 319 | Positive |
| **Morin** | 1 | 153 | 1 | 8 | 30 | 303 | Positive |
| **Hesperetin** | 1 | 153 | 1 | 6.8 | 30 | 303 | Positive |
| **Quercitin** | 1 | 153 | 1 | 8 | 30 | 303 | Positive |
| **Epicatechin** | 1 | 139 | 1 | 5 | 30 | 291 | Positive |
| **Catechin** | 1 | 139 | 1 | 4.2 | 30 | 291 | Positive |
| **Kaempferol** | 1 | 153 | 1 | 8.6 | 30 | 287 | Positive |
| **Luteolin** | 1 | 153 | 1 | 7.9 | 30 | 287 | Positive |
| **Phloretin** | 1 | 107 | 1 | 7.1 | 30 | 275 | Positive |
| **Narigenin** | 1 | 153 | 1 | 8.4 | 30 | 273 | Positive |
| **Genistein** | 1 | 153 | 1 | 8.4 | 30 | 271 | Positive |
| **Apigenin** | 1 | 153 | 1 | 8.4 | 30 | 271 | Positive |
| **Daidzein** | 1 | 137 | 1 | 9.6 | 30 | 255 | Positive |
| **Chalcone** | 1 | 104 | 1 | 11.2 | 30 | 210 | Positive |
