# Supplementary material for: Broad range flavonoid profiling by LC/MS of soybean genotypes contrasting for resistance to Anticarsia gemmatalis (Lepidoptera: Noctuidae)
Source: PLoS One. 2018 Oct 3;13(10):e0205010. doi: 10.1371/journal.pone.0205010 (PMC6169965; doi:10.1371/journal.pone.0205010)
Supplement: S2 Table — (DOCX) [file pone.0205010.s004.docx]

**Table S2**: Transition list used as input for Skyline in the analysis flavonoid classes.

| **Molecule List Name** | **Precursor Charge** | **Product m/z** | **Product charge** | **Precursor RT** | **Precursor CE** | **Precursor m/z** |
| --- | --- | --- | --- | --- | --- | --- |
| Daidzein Class | 1 | 137 | 1 | 6.5 | 30 | 255 |
| Genistein Apigenin Class | 1 | 153 | 1 | 7.25 | 30 | 271 |
| Phloretin Class | 1 | 79 | 1 | 8.33 | 30 | 275 |
| Kaempferol Luteolin Class | 1 | 153 | 1 | 5.58 | 30 | 287 |
| Epicatechin Catechin Class | 1 | 69 | 1 | 0.57 | 30 | 291 |
| Morin Herperentin Quercetin Class | 1 | 229.2 | 1 | 5.23 | 30 | 303 |
| Myricetin Class | 1 | 153 | 1 | 5.65 | 30 | 319 |
| Naringenin Class | 1 | 153 | 1 | 7.34 | 30 | 273 |
