# Supplementary material for: Broad range flavonoid profiling by LC/MS of soybean genotypes contrasting for resistance to Anticarsia gemmatalis (Lepidoptera: Noctuidae)
Source: PLoS One. 2018 Oct 3;13(10):e0205010. doi: 10.1371/journal.pone.0205010 (PMC6169965; doi:10.1371/journal.pone.0205010)
Supplement: S3 Table — (DOCX) [file pone.0205010.s005.docx]

**Table S3:** Transition list used as input in the Skyline analysis for the profiling of the flavonoid compounds in the soybean leaf.

| **Molecule**  **Name** | **Precursor Charge** | **Product m/z** | **Product charge** | **Precursor RT** | **Precursor CE** | **Precursor m/z** |
| --- | --- | --- | --- | --- | --- | --- |
| **Daidzein Class 1** | 1 | 137 | 1 | 7.7 | 30 | 255 |
| **Daidzein Class 1** | 1 | 181 | 1 | 7.7 | 30 | 255 |
| **Daidzein Class 1** | 1 | 153 | 1 | 7.7 | 30 | 255 |
| **Daidzein Class 1** | 1 | 91 | 1 | 7.7 | 30 | 255 |
| **Apigenin Class 2** | 1 | 153 | 1 | 8.55 | 30 | 271 |
| **Apigenin Class 2** | 1 | 145 | 1 | 8.55 | 30 | 271 |
| **Apigenin Class 2** | 1 | 91 | 1 | 8.55 | 30 | 271 |
| **Apigenin Class 2** | 1 | 69 | 1 | 8.55 | 30 | 271 |
| **Genistein Class 2** | 1 | 153 | 1 | 8.44 | 30 | 271 |
| **Genistein Class 2** | 1 | 145 | 1 | 8.44 | 30 | 271 |
| **Genistein Class 2** | 1 | 91 | 1 | 8.44 | 30 | 271 |
| **Genistein Class 2** | 1 | 69 | 1 | 8.44 | 30 | 271 |
| **Phloretin Class 3** | 1 | 150.7 | 1 | 7.11 | 30 | 275 |
| **Phloretin Class 3** | 1 | 107 | 1 | 7.11 | 30 | 275 |
| **Phloretin Class 3** | 1 | 79 | 1 | 7.11 | 30 | 275 |
| **Phloretin Class 3** | 1 | 77 | 1 | 7.11 | 30 | 275 |
| **Luteolin Class 4** | 1 | 153 | 1 | 7.97 | 30 | 287 |
| **Luteolin Class 4** | 1 | 135 | 1 | 7.97 | 30 | 287 |
| **Luteolin Class 4** | 1 | 121 | 1 | 7.97 | 30 | 287 |
| **Luteolin Class 4** | 1 | 69 | 1 | 7.97 | 30 | 287 |
| **Kaempferol Class 4** | 1 | 153 | 1 | 8.73 | 30 | 287 |
| **Kaempferol Class 4** | 1 | 135 | 1 | 8.73 | 30 | 287 |
| **Kaempferol Class 4** | 1 | 121 | 1 | 8.73 | 30 | 287 |
| **Kaempferol Class 4** | 1 | 69 | 1 | 8.73 | 30 | 287 |
| **Catechin Class 5** | 1 | 161 | 1 | 4.26 | 30 | 291 |
| **Catechin Class 5** | 1 | 123 | 1 | 4.26 | 30 | 291 |
| **Catechin Class 5** | 1 | 119.2 | 1 | 4.26 | 30 | 291 |
| **Catechin Class 5** | 1 | 69 | 1 | 4.26 | 30 | 291 |
| **Epicatechin Class 5** | 1 | 161 | 1 | 5.02 | 30 | 291 |
| **Epicatechin Class 5** | 1 | 123 | 1 | 5.02 | 30 | 291 |
| **Epicatechin Class 5** | 1 | 119.2 | 1 | 5.02 | 30 | 291 |
| **Epicatechin Class 5** | 1 | 69 | 1 | 5.02 | 30 | 291 |
| **Quercetin Class 6** | 1 | 229.2 | 1 | 8.05 | 30 | 303 |
| **Quercetin Class 6** | 1 | 153 | 1 | 8.05 | 30 | 303 |
| **Quercetin Class 6** | 1 | 137 | 1 | 8.05 | 30 | 303 |
| **Quercetin Class 6** | 1 | 89 | 1 | 8.05 | 30 | 303 |
| **Hesperentin Class 6** | 1 | 229.2 | 1 | 7.94 | 30 | 303 |
| **Hesperentin Class 6** | 1 | 153 | 1 | 7.94 | 30 | 303 |
| **Hesperentin Class 6** | 1 | 137 | 1 | 7.94 | 30 | 303 |
| **Hesperentin Class 6** | 1 | 89 | 1 | 7.94 | 30 | 303 |
| **Morin Class 6** | 1 | 229.2 | 1 | 7.83 | 30 | 303 |
| **Morin Class 6** | 1 | 153 | 1 | 7.83 | 30 | 303 |
| **Morin Class 6** | 1 | 137 | 1 | 7.83 | 30 | 303 |
| **Morin Class 6** | 1 | 89 | 1 | 7.83 | 30 | 303 |
| **Myricetin Class 7** | 1 | 245 | 1 | 7.39 | 30 | 319 |
| **Myricetin Class 7** | 1 | 217 | 1 | 7.39 | 30 | 319 |
| **Myricetin Class 7** | 1 | 164.8 | 1 | 7.39 | 30 | 319 |
| **Myricetin Class 7** | 1 | 153 | 1 | 7.39 | 30 | 319 |
| **Naringenin Class 8** | 1 | 153 | 1 | 8.54 | 30 | 273 |
| **Naringenin Class 8** | 1 | 147 | 1 | 8.54 | 30 | 273 |
| **Naringenin Class 8** | 1 | 119 | 1 | 8.54 | 30 | 273 |
| **Naringenin Class 8** | 1 | 91 | 1 | 8.54 | 30 | 273 |
