# Supplementary material for: Broad range flavonoid profiling by LC/MS of soybean genotypes contrasting for resistance to Anticarsia gemmatalis (Lepidoptera: Noctuidae)
Source: PLoS One. 2018 Oct 3;13(10):e0205010. doi: 10.1371/journal.pone.0205010 (PMC6169965; doi:10.1371/journal.pone.0205010)
Supplement: S4 Table — (DOCX) [file pone.0205010.s006.docx]

**Table** **S4**: Intensities of the characterized flavonoids in the non-target analysis for each genotypes and treatment used as input in the MetaboAnalyst platform.

| **Name** | 105 AP T1A1 | 105 AP T1A2 | 105 AP T1B1 | 105 AP T1B2 | 105 AP T2A1 | 105 AP T2A2 | 105 AP T2B1 | 105 AP T2B2 | IAC 17 T1A1 | IAC 17 T1A2 | IAC 17 T1B1 | IAC 17 T1B2 | IAC 17 T2A1 | IAC 17 T2A2 | IAC 17 T2B1 | IAC 17 T2B2 |
| --- | --- | --- | --- | --- | --- | --- | --- | --- | --- | --- | --- | --- | --- | --- | --- | --- |
| **sample** | 105AP T1 | 105AP T1 | 105AP T1 | 105AP T1 | 105AP T2 | 105AP T2 | 105AP T2 | 105AP T2 | IAC17 T1 | IAC17 T1 | IAC17 T1 | IAC17 T1 | IAC17 T2 | IAC17 T2 | IAC17 T2 | IAC17 T2 |
| **Dadzein-4.5** | 496 | 551 | 623 | 557 | 651 | 484 | 555 | 530 | 523 | 399 | 563 | 766 | 300 | 243 | 332 | 252 |
| **Daidzein-5.6** | 2787 | 1717 | 2761 | 2422 | 5703 | 5392 | 5457 | 5278 | 2505 | 2490 | 2616 | 2853 | 4194 | 2957 | 4559 | 3366 |
| **Daidzein-6.2** | 20122 | 15102 | 21479 | 18901 | 14600 | 46593 | 36856 | 49377 | 3619 | 5431 | 5006 | 5970 | 4826 | 6310 | 4648 | 5457 |
| **Daidzein-6.5** | NA | NA | NA | NA | NA | NA | NA | NA | 3619 | 5431 | 5006 | 5970 | 4826 | 6310 | 4648 | 5457 |
| **Daidzein-7.6** | NA | NA | NA | NA | NA | NA | NA | NA | 829 | 980 | 1090 | 1090 | 1561 | 2075 | 1650 | 2010 |
| **Genistein-5.3** | 1819 | 1523 | 2315 | 1886 | 1703 | 1566 | 1632 | 1627 | 443 | 551 | 573 | 726 | 480 | 622 | 538 | 581 |
| **Genistein-6.4** | 1728 | 1295 | 2435 | 1819 | 1822 | 1431 | 1631 | 1640 | 377 | 495 | 720 | 1287 | 2188 | 1324 | 2526 | 2000 |
| **Genistein-6.7** | NA | NA | NA | NA | NA | NA | NA | NA | 912 | 1292 | 1029 | 885 | 4219 | 1324 | 3880 | 1400 |
| **Apigenin-6.7** | 17864 | 16684 | 22950 | 19166 | 16198 | 18951 | 18240 | 19571 | NA | NA | NA | NA | NA | NA | NA | NA |
| **Genistein-7.2** | NA | NA | NA | NA | NA | NA | NA | NA | 29880 | 16832 | 22156 | 19756 | 40287 | 45754 | 43452 | 46134 |
| **Apigenin-7.2** | 101020 | 90390 | 120153 | 103854 | 168844 | 155626 | 162243 | 162260 | NA | NA | NA | NA | NA | NA | NA | NA |
| **Genistein-8.6** | NA | NA | NA | NA | NA | NA | NA | NA | 1387 | 1994 | 1962 | 2505 | 1712 | 7183 | 1928 | 3168 |
| **Apigenin-8.6** | 6659 | 22361 | 8706 | 12575 | 3436 | 5818 | 4807 | 5167 | NA | NA | NA | NA | NA | NA | NA | NA |
| **Quercetin-5.2** | 1694 | 287 | 2156 | 1379 | 1627 | 1728 | 1713 | 1784 | 32854 | 30800 | 33179 | 35884 | 19518 | 19578 | 20206 | 19991 |
| **Quercetin-6.2** | 164 | 168 | 201 | 177 | 155 | 186 | 182 | 205 | 24196 | 23152 | 24904 | 27364 | 17008 | 17668 | 17555 | 17948 |
| **Quercetin-5.5** | 386 | 271 | 432 | 363 | 372 | 321 | 364 | 401 | 4410 | 4606 | 4614 | 4826 | 2323 | 2346 | 2291 | 1868 |
| **Quercetin-7.1** | 492 | 243 | 581 | 383 | 142 | 121 | 131 | 131 | 295 | 450 | 302 | 614 | 172 | 244 | 117 | 196 |
| **Quercetin-7.6** | 33 | 58 | 34 | 42 | 144 | 178 | 161 | 161 | 60 | 25 | 41 | 38 | 305 | 278 | 339 | 373 |
| **Kaempferol-5.6** | 452751 | 415116 | 506208 | 458025 | 416431 | 426100 | 429746 | 446709 | 228314 | 235439 | 236761 | 246531 | 214238 | 219520 | 210521 | 224124 |
| **Kaempferol-5.9** | 186514 | 172484 | 198907 | 185968 | 140366 | 139823 | 142218 | 146466 | 138892 | 151602 | 151880 | 165146 | 166228 | 176144 | 163839 | 175537 |
| **Kaempferol-6.2** | 6769 | 6991 | 6897 | 5868 | 12648 | 8371 | 13311 | 8932 | 1357 | 1579 | 2396 | 985 | 7804 | 8527 | 10638 | 12009 |
| **Kaempferol-6.7** | 105675 | 109251 | 111831 | 108919 | 73266 | 90019 | 85184 | 92268 | 120691 | 156742 | 146156 | 161037 | 121875 | 137677 | 105497 | 118648 |
| **Kaempferol-7.9** | 1669 | 1834 | 1942 | 1815 | 1851 | 515 | 1997 | 3626 | 11590 | 14752 | 14745 | 17894 | 13005 | 13966 | 15878 | 18093 |
| **Rutin** | 805 | 945 | 799 | 849 | 701 | 923 | 840 | 895 | 156558 | 168880 | 168758 | 180836 | 109076 | 118272 | 110006 | 112806 |
| **Naringin** | 10 | 24 | 44 | 26 | 15 | 23 | 20 | 21 | 82 | 70 | 64 | 41 | 37 | 23 | 13 | 31 |
| **Luteolin** | 1274 | 664 | 463 | 800 | 436 | 328 | 283 | 384 | 17428 | 21924 | 22522 | 28214 | 11844 | 15458 | 16612 | 19649 |
| **Naringenin** | 546 | 710 | 519 | 591 | 331 | 1779 | 1248 | 1636 | 299 | 384 | 364 | 410 | 960 | 1229 | 823 | 977 |
| **Kaempferol** | 1313 | 1359 | 685 | 1119 | 1067 | 1720 | 1459 | 1591 | 305 | 372 | 370 | 434 | 677 | 1139 | 952 | 1388 |
| **Daidzein** | 42 | 41 | 11 | 31 | 35 | 13 | 21 | 14 | 23 | 25 | 26 | 31 | 48 | 56 | 50 | 39 |
| **Quercetin** | 185 | 57 | 104 | 115 | 266 | 78 | 125 | 30 | 97 | 116 | 101 | 89 | 76 | 113 | 69 | 71 |
| **Genistein** | 9476 | 15396 | 9453 | 11441 | 6208 | 9073 | 7860 | 8299 | 2286 | 3344 | 3269 | 4177 | 3158 | 11336 | 3674 | 5195 |
| **Apigenin** | 9266 | 14883 | 9309 | 11152 | 6155 | 9073 | 7842 | 8299 | 2286 | 3344 | 3269 | 4177 | 3158 | 11336 | 3674 | 5253 |
