# Supplementary material for: Broad range flavonoid profiling by LC/MS of soybean genotypes contrasting for resistance to Anticarsia gemmatalis (Lepidoptera: Noctuidae)
Source: PLoS One. 2018 Oct 3;13(10):e0205010. doi: 10.1371/journal.pone.0205010 (PMC6169965; doi:10.1371/journal.pone.0205010)
Supplement: S5 Table — The data were used for statistical and clustering analysis using the MetaboAnalyst platform. (DOCX) [file pone.0205010.s007.docx]

**Table S5**: Results of the statistical analysis by ANOVA using the MetaboAnalyst platform.

|  | f.value | p.value | -log10(p) | FDR | Fisher's LSD |
| --- | --- | --- | --- | --- | --- |
| Rutin | 19929 | 1.9151e-22 | 21718 | 5.3622e-21 | IAC17 T1 - 105AP T1; IAC17 T2 - 105AP T1; IAC17 T1 - 105AP T2; IAC17 T2 - 105AP T2; IAC17 T1 - IAC17 T2 |
| Quercetin-6.2 | 1162.5 | 4.7613e-15 | 14322 | 6.6658e-14 | IAC17 T1 - 105AP T1; IAC17 T2 - 105AP T1; IAC17 T1 - 105AP T2; IAC17 T2 - 105AP T2; IAC17 T1 - IAC17 T2 |
| Apigenin-6.7 | 686.92 | 1.1014e-13 | 12958 | 8.4937e-13 | 105AP T1 - IAC17 T1; 105AP T1 - IAC17 T2; 105AP T2 - IAC17 T1; 105AP T2 - IAC17 T2 |
| Quercetin-5.2 | 675.86 | 1.2134e-13 | 12916 | 8.4937e-13 | IAC17 T1 - 105AP T1; IAC17 T2 - 105AP T1; IAC17 T1 - 105AP T2; IAC17 T2 - 105AP T2; IAC17 T1 - IAC17 T2 |
| Apigenin-7.2 | 642.11 | 1.6467e-13 | 12783 | 8.5344e-13 | 105AP T2 - 105AP T1; 105AP T1 - IAC17 T1; 105AP T1 - IAC17 T2; 105AP T2 - IAC17 T1; 105AP T2 - IAC17 T2 |
| Quercetin-5.5 | 620.36 | 2.0222e-13 | 12694 | 8.5344e-13 | IAC17 T1 - 105AP T1; IAC17 T2 - 105AP T1; IAC17 T1 - 105AP T2; IAC17 T2 - 105AP T2; IAC17 T1 - IAC17 T2 |
| Luteolin-5.6 | 614.8 | 2.1336e-13 | 12671 | 8.5344e-13 | 105AP T1 - IAC17 T1; 105AP T1 - IAC17 T2; 105AP T2 - IAC17 T1; 105AP T2 - IAC17 T2 |
| Daidzein-7.6 | 339.69 | 7.2514e-12 | 11.14 | 2.538e-11 | IAC17 T1 - 105AP T1; IAC17 T2 - 105AP T1; IAC17 T1 - 105AP T2; IAC17 T2 - 105AP T2; IAC17 T2 - IAC17 T1 |
| Luteolin-5.9 | 248.12 | 4.6454e-11 | 10333 | 1.4452e-10 | 105AP T1 - 105AP T2; 105AP T1 - IAC17 T1; IAC17 T2 - 105AP T1; IAC17 T1 - 105AP T2; IAC17 T2 - 105AP T2; IAC17 T2 - IAC17 T1 |
| Daidzein-6.5 | 189.63 | 2.2589e-10 | 9.6461 | 6.3248e-10 | IAC17 T1 - 105AP T1; IAC17 T2 - 105AP T1; IAC17 T1 - 105AP T2; IAC17 T2 - 105AP T2; IAC17 T2 - IAC17 T1 |
| Genistein-7.2 | 158.43 | 6.4705e-10 | 9.1891 | 1.647e-09 | IAC17 T1 - 105AP T1; IAC17 T2 - 105AP T1; IAC17 T1 - 105AP T2; IAC17 T2 - 105AP T2; IAC17 T2 - IAC17 T1 |
| Quercetin-7.6 | 112.85 | 4.6483e-09 | 8.3327 | 1.0846e-08 | 105AP T2 - 105AP T1; IAC17 T2 - 105AP T1; 105AP T2 - IAC17 T1; IAC17 T2 - 105AP T2; IAC17 T2 - IAC17 T1 |
| Luteolin-7.9 | 104.14 | 7.391e-09 | 8.1313 | 1.5431e-08 | IAC17 T1 - 105AP T1; IAC17 T2 - 105AP T1; IAC17 T1 - 105AP T2; IAC17 T2 - 105AP T2 |
| Genistein-5.3 | 102.5 | 8.0987e-09 | 8.0916 | 1.5431e-08 | 105AP T1 - IAC17 T1; 105AP T1 - IAC17 T2; 105AP T2 - IAC17 T1; 105AP T2 - IAC17 T2 |
| Luteolin | 102.14 | 8.2664e-09 | 8.0827 | 1.5431e-08 | IAC17 T1 - 105AP T1; IAC17 T2 - 105AP T1; IAC17 T1 - 105AP T2; IAC17 T2 - 105AP T2; IAC17 T1 - IAC17 T2 |
| Luteolin-6.7 | 56145 | 2.4641e-07 | 6.6083 | 4.3122e-07 | 105AP T1 - 105AP T2; IAC17 T1 - 105AP T1; IAC17 T2 - 105AP T1; IAC17 T1 - 105AP T2; IAC17 T2 - 105AP T2; IAC17 T1 - IAC17 T2 |
| Daidzein-5.6 | 22485 | 3.2472e-05 | 4.4885 | 5.3483e-05 | 105AP T2 - 105AP T1; IAC17 T2 - 105AP T1; 105AP T2 - IAC17 T1; 105AP T2 - IAC17 T2; IAC17 T2 - IAC17 T1 |
| Daidzein-6.2 | 15177 | 0.00021882 | 3.6599 | 0.00034038 | 105AP T2 - 105AP T1; 105AP T1 - IAC17 T1; 105AP T1 - IAC17 T2; 105AP T2 - IAC17 T1; 105AP T2 - IAC17 T2 |
| Kaempferol | 11648 | 0.00072288 | 3.1409 | 0.0010653 | 105AP T1 - IAC17 T1; 105AP T2 - IAC17 T1; IAC17 T2 - IAC17 T1 |
| Apigenin-8.6 | 9.5239 | 0.0016958 | 2.7706 | 0.0023333 | 105AP T1 - 105AP T2; 105AP T1 - IAC17 T1; 105AP T1 - IAC17 T2 |
| [Quercetin-7.1](http://www.metaboanalyst.ca/faces/Secure/details/FeatureDetailsView.xhtml) | 10.034 | 0.0013667 | 2.8643 | 0.0019816 | 105AP T1 - 105AP T2; 105AP T1 - IAC17 T2; IAC17 T1 - 105AP T2; IAC17 T1 - IAC17 T2 |
| Genistein-6.7 | 9.4512 | 0.00175 | 2757 | 0.0023333 | IAC17 T2 - 105AP T1; IAC17 T2 - 105AP T2; IAC17 T2 - IAC17 T1 |
| Genistein-6.4 | 9.2083 | 0.0019459 | 2.7109 | 0.0024767 | 105AP T1 - IAC17 T1; 105AP T2 - IAC17 T1; IAC17 T2 - 105AP T2; IAC17 T2 - IAC17 T1 |
| Genistein-8.6 | 7.8813 | 0.0036001 | 2.4437 | 0.0043827 | IAC17 T2 - 105AP T1; IAC17 T2 - 105AP T2 |
| [Kaempferol-6.2](http://www.metaboanalyst.ca/faces/Secure/details/FeatureDetailsView.xhtml) | 23.44 | 2.6323E-5 | 4.5797 | 4.6452E-5 | 105AP T2 - 105AP T1; 105AP T1 - IAC17 T1; IAC17 T2 - 105AP T1; 105AP T2 - IAC17 T1; IAC17 T2 - IAC17 T1 |
| Dadzein-4.5 | 6.89 | 0.0059582 | 2.2249 | 0.0069513 | 105AP T1 - IAC17 T2; 105AP T2 - IAC17 T2; IAC17 T1 - IAC17 T2 |
| Naringenin | 6.6865 | 0.0066429 | 2.1776 | 0.00744 | 105AP T2 - 105AP T1; IAC17 T2 - 105AP T1; 105AP T2 - IAC17 T1; IAC17 T2 - IAC17 T1 |
| Genistein | 5.6616 | 0.011853 | 1.9262 | 0.012765 | 105AP T1 - IAC17 T1; 105AP T1 - IAC17 T2; 105AP T2 - IAC17 T1 |
| Apigenin | 5.4642 | 0.013337 | 1.8749 | 0.013831 | 105AP T1 - IAC17 T1; 105AP T1 - IAC17 T2; 105AP T2 - IAC17 T1 |
